# Supplementary material for: Stepwise π-extension of double [5]helicene diimides to planar nanographene diimides
Source: Commun Chem. 2025 Nov 18;8:353. doi: 10.1038/s42004-025-01743-4 (PMC12627696; doi:10.1038/s42004-025-01743-4)
Supplement: Supplementary file 2 — Supporting Information [file 42004_2025_1743_MOESM2_ESM.pdf]

Supporting Information

Stepwise π-Extension of double [5]Helicene Diimides to Planar Nanographene Diimides

Vikas Sharma[a], Jacob Isaac[b,c], Anmol Thanai[a], Kieran Richards[b,c], Daniel T. W. Toolan[d], George F. S. Whitehead[a], Emrys W. Evans[b,c] and Ashok Keerthi[a,e,f]*

[a]Department of Chemistry, School of Natural Sciences, The University of Manchester, Oxford Road, M13 9PL, UK.

[b]Department of Chemistry, Swansea University, Singleton Park, Swansea SA2 8PP, UK.

[c]Centre for Integrative Semiconductor Materials, Swansea University, Fabian Way, Swansea SA1 8EN, UK.

[d]Department of Materials, School of Natural Sciences, The University of Manchester, Booth Street East, Manchester, M13 9SS, UK.

[e]Photon Science Institute, The University of Manchester, Manchester, M13 9PL, UK.

[f]National Graphene Institute, The University of Manchester, Booth Street East, Manchester, M13 9SS, UK.

*Corresponding author: ashok.keerthi@manchester.ac.uk

Contents:

1. Synthesis page S2
2. ^{1}H-NMR, ^{13}C-NMR & HRMS spectra of synthesized compounds page S10
3. Cyclic voltammetry data page S22
4. Density functional theory calculations page S23
5. Single-crystals crystallographic data page S24
6. Time-resolved photoluminescence data page S26
7. Grazing Incidence Wide Angle Scattering Data page S28
8. Chiral HPLC chromatograms page S29
9. References page S29

1. Synthesis

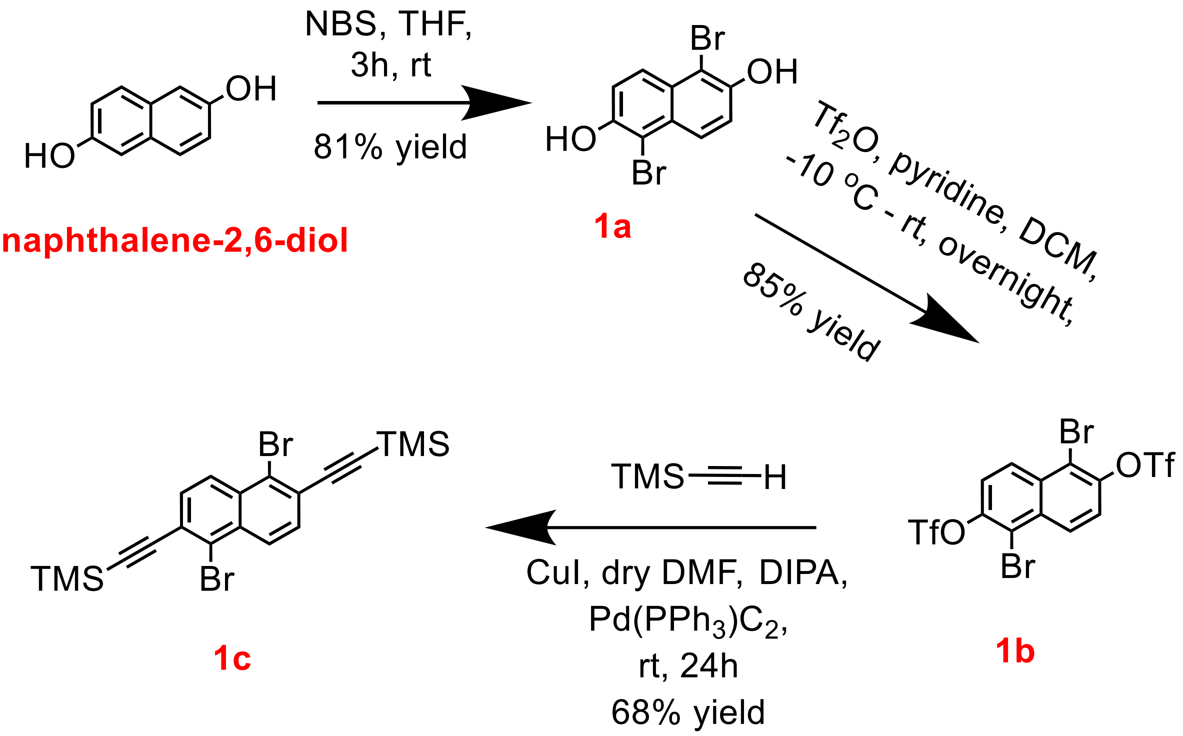

Scheme S1. Synthesis of compound **1c**.

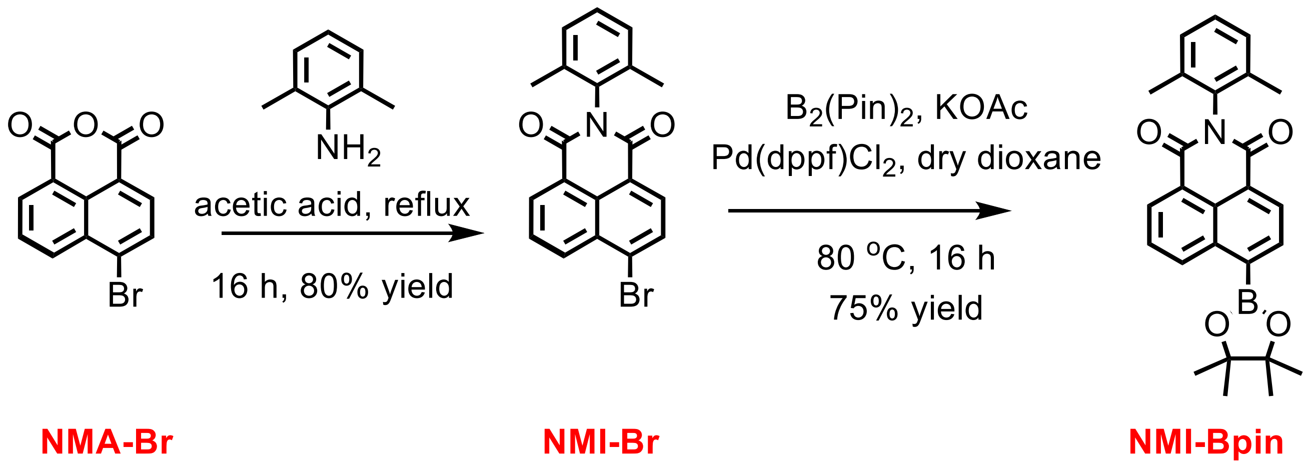

Scheme S2. Synthesis of **NMI-Bpin**.

1,5-dibromonaphthalene-2,6-diol (1a)

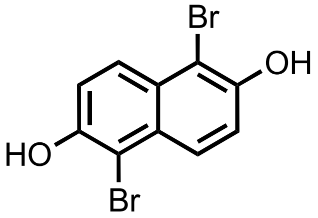

Under N_2 atmosphere compound naphthalene-2, 6-diol (1 g, 6.24 mmol) was added to a round bottom flask and 50 ml of dry THF was added. The flask was covered with aluminum foil. After every 5-10 min NBS (2.4 g, 13.73 mmol) was added portion wise at room temperature. The reaction was stirred further 3h at rt. In the reaction mixture 100 ml water was added and extracted with EA. (1.6 g, 5.054 mmol, yield 81%,) **^{1}H NMR** (500 MHz, DMSO-D_6) δ 10.40 (s, 2H), 7.94 (d, *J* = 9.1 Hz, 2H), 7.33 (d, *J* = 9.1 Hz, 2H). **^{13}C NMR** (126 MHz, DMSO-d_6) δ 104.92, 119.64, 125.98, 127.88, 150.61, 179.41.

1,5-dibromonaphthalene-2,6-diyl bis(trifluoromethanesulfonate) (1b)

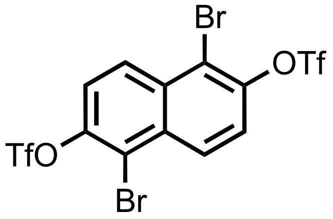

Compound 1a (1.4 g, 4.40 mmol) and Pyridine (1.4 ml, 17.6 mmol) were added to a round-bottom flask in a nitrogen environment and dissolved in 100 mL of DCM. Trifluoromethanesulfonic anhydride (3 mL, 17.6 mmol) was added to the reaction mixture after it had been cooled to -10 °C. Further the reaction mixture was stirred overnight at room temperature in a nitrogen environment. DCM was used to extract the reaction after it had been quenched with water and a 1M HCl solution. After being cleaned with brine and saturated aqueous $NaHCO_3$ solution, the mixed organic layers were dried. Further crude compound was dissolved in DCM and precipitated were collected by adding methanol provided the pure product **1b** (2.18 g, 3.74 mmol, 85%). **^{1}H NMR** (500 MHz, $CDCl_3$) δ 8.46 (d, *J* = 9.2 Hz, 2H), 7.65 (d, *J* = 9.3 Hz, 2H). **^{13}C NMR** (126 MHz, $CDCl_3$) δ 116.79, 122.88, 130.04, 132.62, 146.61.

((1,5-dibromonaphthalene-2,6-diyl) bis(ethyne-2,1-diyl))bis(trimethylsilane) (1c)

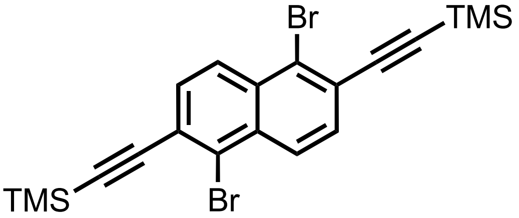

Sparged DMF (15 mL), DIPA (2.6 mL), and TMSA (1.7 mL, 12.36 mmol) were mixed into an oven-dried flask containing $PdCl_2(PPh_3)_2$ (96 mg, 0.13 mmol), CuI (26 mg, 0.13 mmol), and 1b (800 mg, 1.37 mmol). The reaction was stirred at room temperature for two days and subsequently quenched with a 5% aqueous solution of NH_4Cl. Additional excess water was introduced, and the precipitates were collected through vacuum filtration. The crude product underwent purification via column chromatography using a Hexane: DCM (10:1) eluent, resulting in an orange solid (450 g, 0.94 mmol, yield 68 %). **^{1}H NMR** (400 MHz, $CDCl_3$) δ 8.21 (d, *J* = 8.7 Hz, 2H), 7.59 (d, *J* = 10.1 Hz, 2H), 0.32 (s, 18H). **^{13}C NMR** (126 MHz, $CDCl_3$) δ -0.04, 102.45, 103.80, 124.91, 126.74, 127.49, 130.99, 132.44.

6-bromo-2-(2,6-dimethylphenyl)-1*H*-benzo[*de*]isoquinoline-1,3(2*H*)-dione (NMI-Br)

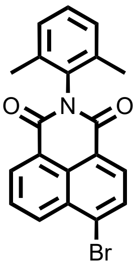

4-Bromo-1,8-naphthalic anhydride (NMA-Br, 2 g, 7.22 mmol) was dissolved in glacial acetic acid (30 mL) under nitrogen atmosphere followed by 2,6-dimethylaniline (1.7 g, 14.42 mmol). The reaction mixture was stirred overnight at 130 °C. After this time, the solution was cooled and poured into ice water to form a precipitate, followed by filtration afforded a off white product (2.2 g, 5.78 mmol 80%) **^{1}H NMR** (400 MHz, $CDCl_3$) δ 8.73 (dd, J = 7.3, 1.2 Hz, 1H), 8.67 (d, J = 8.5 Hz, 1H), 8.49 (d, J = 7.9 Hz, 1H), 8.10 (d, J = 7.8 Hz, 1H), 7.91 (dd, J = 8.5, 7.3 Hz, 1H), 7.29 (dd, J = 8.5, 6.5 Hz, 1H), 7.22 (d, J = 7.5 Hz, 2H), 2.14 (s, 6H).

2-(2,6-dimethylphenyl)-6-(4,4,5,5-tetramethyl-1,3,2-dioxaborolan-2-yl)-1*H*-benzo[*de*]isoquinoline-1,3(2*H*)-dione (NMI-Bpin)

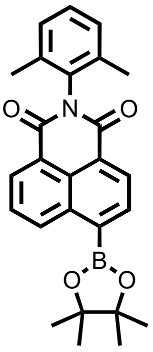

Under argon atmosphere NMI-Br (1 g, 2.62 mmol), B_2Pin_2 (1.01 g, 3.94 mmol), and KOAc (514 mg, 5.24 mmol) were dissolved in dry dioxane (40 ml). After degassing with nitrogen for 30 minutes, the catalyst Pd(dppf)Cl_2 (190 mg) was added and further degassing with nitrogen for 30 min, the mixture was heated at 80 °C for 24 h. The resulting mixture was filtered over celite and washed with of DCM. The crude product was purified by column chromatography with Hexane: EA (10: 1) as eluent yielding a yellow solid (840 mg, 1.96 mmol, yield 75 %). **APCI-HRMS**: m/z 428.2032 calculated 427.1955, **^{1}H NMR** (500 MHz, $CDCl_3$) δ 9.21 (dd, J = 8.4, 1.2 Hz, 1H), 8.67 (dd, J = 7.2, 1.2 Hz, 1H), 8.63 (d, J = 7.2 Hz, 1H), 8.35 (d, J = 7.2 Hz, 1H), 7.84 (dd, J = 8.5, 7.2 Hz, 1H), 7.31 – 7.26 (m, 1H), 7.22 (d, J = 6.9 Hz, 2H), 2.14 (s, 6H), 1.47 (s, 12H). **^{13}C NMR** (126 MHz, $CDCl_3$) δ 18.02, 25.13, 84.79, 122.74, 124.87, 127.31, 128.63, 128.68, 128.94, 130.35, 131.46, 134.05, 135.55, 135.65, 135.67, 135.97, 163.74, 163.77.

6,6'-(2,6-bis((trimethylsilyl)ethynyl) naphthalene-1,5-diyl) bis(2-(2,6-dimethylphenyl)-1*H*-benzo[*de*]isoquinoline-1,3(2*H*)-dione) (2)

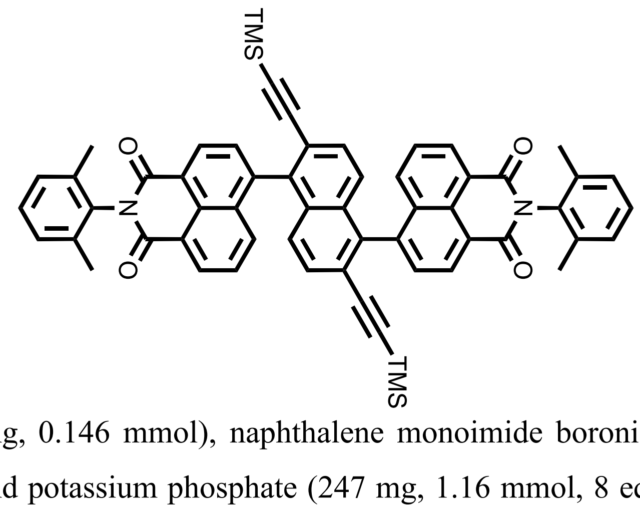

Compound **1c** (70 mg, 0.146 mmol), naphthalene monoimide boronic ester (162 mg, 0.38 mmol, 2.6 equiv.), and potassium phosphate (247 mg, 1.16 mmol, 8 equiv.) were mixed in a solution of toluene (7.5 mL), ethanol (1.5 mL), and water (1.5 mL). After the reaction mixture

was bubbled under nitrogen for 20 mins, catalyst tetrakis (triphenylphosphine) palladium (0) (34 mg, 0.03 mmol, 20 mol%) was added. The reaction mixture was heated to 100 °C and stirred for 24 h. The reaction mixture was poured into water and extracted with DCM three times and dried over $MgSO_4$. After removing the solvent under reduced pressure, the crude product was purified by column chromatography with Hexane: EA (10: 2) as eluent yielding a light-yellow solid (95 mg, 0.103 mmol, yield 71 %). **APCI-HRMS**: m/z 918.3304 calculated 918.3309, **^{1}H NMR** (500 MHz, $CDCl_3$) δ 8.86 (dd, J = 7.3, 3.8 Hz, 2H), 8.74 (t, J = 1.5 Hz, 2H), 7.96 – 7.79 (m, 4H), 7.71 (td, J = 8.8, 7.1 Hz, 2H), 7.59 – 7.49 (m, 4H), 7.33 (t, J = 7.5 Hz, 2H), 7.27 (d, J = 17.1 Hz, 4H), 2.26 (s, 6H), 2.16 (s, 6H), -0.31 (d, J = 4.3 Hz, 18H). **^{13}C NMR** (126 MHz, $CDCl_3$) δ -0.72, -0.70, 17.81, 18.14, 102.29, 103.67, 122.69, 123.10, 126.94, 127.30, 128.74, 129.07, 129.25, 129.47, 129.57, 131.33, 131.44, 131.98, 134.04, 135.50, 135.79, 140.54, 144.20, 163.48, 163.65.

6,6'-(2,6-diethynylnaphthalene-1,5-diyl)bis(2-(2,6-dimethylphenyl)-1*H*-benzo[*de*]isoquinoline-1,3(2*H*)-dione) (3)

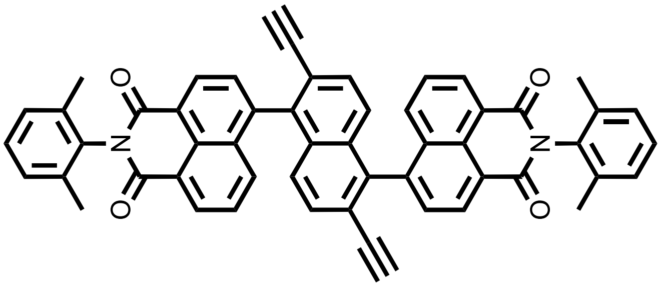

To a solution of the TMS-protected compound **2** (200 mg, 0.22 mmol), in THF and methanol having ratio 4:1 respectively, was added K_2CO_3 (120 mg, 0.87 mmol, 4eq.) and stirred for 4h at room temperature. The reaction mixture was poured into water and extracted with DCM three and dried over $MgSO_4$. After removing the solvent under reduced pressure which provided the pure product **3** (157 mg, 0.20 mmol, 92%). **APCI-HRMS**: m/z 774.2513 calculated 774.2519, **^{1}H NMR** (500 MHz, $CDCl_3$) δ 8.85 (dd, J = 7.4, 5.8 Hz, 2H), 8.73 (ddd, J = 8.8, 7.2, 1.2 Hz, 2H), 7.97 – 7.83 (m, 3H), 7.78 – 7.67 (m, 3H), 7.61 (d, J = 8.8 Hz, 2H), 7.37 (dd, J = 8.7, 4.7 Hz, 2H), 7.35 – 7.31 (m, 2H), 7.27 (d, J = 8.5 Hz, 4H), 2.94 (d, J = 2.9 Hz, 2H), 2.25 (d, J = 4.1 Hz, 12H). **^{13}C NMR** (126 MHz, $CDCl_3$) δ 18.21, 18.22, 81.99, 83.79, 121.78, 123.06, 123.35, 127.21, 127.54, 128.73, 128.75, 129.09, 129.21, 129.76, 130.42, 131.25, 131.37, 132.06, 132.15, 132.73, 134.00, 135.64, 135.78, 140.05, 143.35, 163.47, 163.60.

Synthesis of S-NMI

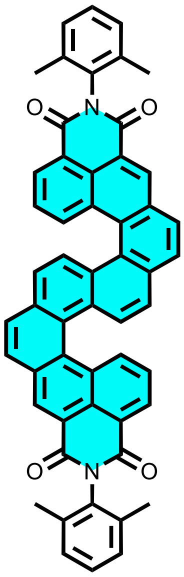

Method 1: To a solution of **1** (50 mg, 0.064 mmol) in dry and degassed toluene (20 mL) $PtCl_2$ (2.6 mg, 0.009 mmol, 15 mol%) was added. The mixture was allowed to stir at 80 °C for two days. The mixture was filtered through Celite, and the residue was washed with DCM. After removal of the solvents under reduced pressure, the crude product was purified by column chromatography using flash silica gel with Hexane: EA (5:1) as eluent to yield a yellow solid (25.8 mg, 0.033 mmol, 52 %).

Method 2: To a solution of **1** (90 mg, 0.12 mmol) in dry and degassed toluene (20 mL) $PtCl_2$ (4.6 mg, 0.02 mmol, 15 mol%) was added. The mixture was allowed to stir at 120 °C for 6h in microwave reactor. The mixture was filtered through Celite, and the residue was washed with DCM. After removal of the solvents under reduced pressure, the crude product was purified by column chromatography using flash silica gel with Hexane: EA (5:1) as eluent to yield a yellow solid (65 mg, 0.083 mmol, 70 %). **APCI-HRMS**: m/z 774.2513 calculated 774.2519, **^{1}H NMR** (400 MHz, $CDCl_3$) δ 9.26 (dd, J = 8.6, 1.2 Hz, 2H), 9.22 (s, 2H), 8.74 (dd, J = 7.3, 1.1 Hz, 2H), 8.55 (d, J = 8.8 Hz, 2H), 8.21 (d, J = 8.3 Hz, 2H), 8.07 (d, J = 8.3 Hz, 2H), 7.79 – 7.66 (m, 2H), 7.38 – 7.32 (m, 1H), 7.29 (d, J = 7.3 Hz, 3H), 2.30 (s, 3H), 2.24 (s, 3H). **^{13}C NMR** (101 MHz, $CDCl_3$) δ 18.21, 29.85, 121.46, 123.10, 125.37, 126.15, 126.89, 127.93, 128.76, 129.11, 129.28, 129.74, 130.19, 130.33, 130.96, 131.82, 131.91, 134.30, 134.34, 134.79, 135.62, 135.85, 163.48, 163.97.

Synthesis of C-PMI

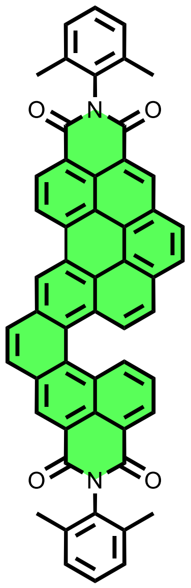

To a solution of 34 mg (0.26mmol, 10 eq.) of $AlCl_3$ in 6 mL of anhydrous chlorobenzene under nitogen atmosphere, compound S-NMI (20 mg, 0.026 mmol) was added in a 50 ml pressure tube, and the mixture was heated at 110 °C for 18 hours. The reaction mixture was poured into water and extracted with DCM three times and dried over $MgSO_4$. After removing the solvent under reduced pressure. The crude product was purified by column chromatography with Hexane: EA (10: 2) as eluent yielding a orange solid (11 mg, 0.014 mmol, yield 55 %). **APCI-HRMS**: m/z 773.2435 calculated 772.2362, **^{1}H NMR** (500 MHz, $CDCl_3$) δ 9.64 (s, 1H), 9.52 (s, 1H), 9.42 (d, J = 8.3 Hz, 1H), 9.29 (s, 1H), 9.17 (d, J = 8.1 Hz, 1H), 8.93 (dd, J = 8.7, 6.1 Hz, 2H), 8.77 – 8.69 (m, 2H), 8.58 (d, J = 8.3 Hz, 1H), 8.49 (d, J = 8.3 Hz, 1H), 8.34 (d, J = 8.4 Hz, 1H), 8.19 (d, J = 9.1 Hz, 1H), 7.69 – 7.62 (m, 1H), 7.38 – 7.28 (m, 6H), 2.28 (dd, J = 25.2, 21.5 Hz, 12H). **^{13}C NMR** (176 MHz, $CDCl_3$) δ 18.15, 18.19, 18.22, 29.85, 120.82, 121.02, 121.45, 121.59, 122.03, 122.15, 122.47, 123.98, 125.86, 125.96, 126.14, 126.88, 127.42, 128.20, 128.61, 128.77, 129.08, 129.14, 129.88, 131.34, 131.75, 131.95, 132.08, 132.19, 132.32, 133.97, 134.25, 135.02, 135.48, 135.70, 135.80, 140.88, 141.97, 142.93, 163.30, 163.44, 163.61, 163.90.

Synthesis of DB-TDI

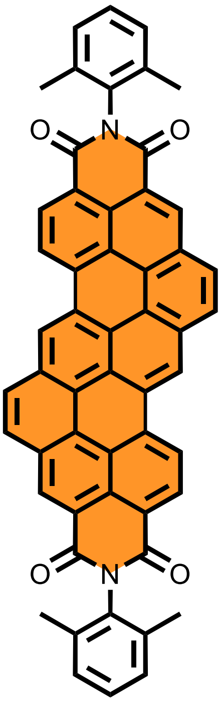

To a solution of 69 mg (0.52 mmol, 20 eq.) of $AlCl_3$ in 6 mL of anhydrous chlorobenzene under oxygen atmosphere, compound S-NMI (20 mg, 0.026 mmol) was added in a 50 ml pressure tube, and the mixture was heated at 130 °C for 18 hours. The reaction mixture was poured into water and extracted with DCM three times and dried over $MgSO_4$. After removing the solvent under reduced pressure. The crude product was purified by column chromatography with Hexane: EA (10: 4) as eluent yielding a orange solid (12 mg, 0.015 mmol, yield 60 %). **APCI-HRMS**: m/z 770.2200 calculated 770.2206, **^{1}H NMR** (400 MHz, 1,1,2,2-Tetrachloroethane-d_2) δ 9.65 (s, 2H), 9.43 (s, 2H), 9.32 (s, 2H), 9.11 (d, J = 8.0 Hz, 2H), 8.68 (d, J = 13.9 Hz, 4H), 7.48 – 7.43 (m, 2H), 7.38 (d, J = 7.6 Hz, 4H), 2.35 (s, 12H).

2. ^{1}H-NMR, ^{13}C-NMR and HRMS of synthesized compounds

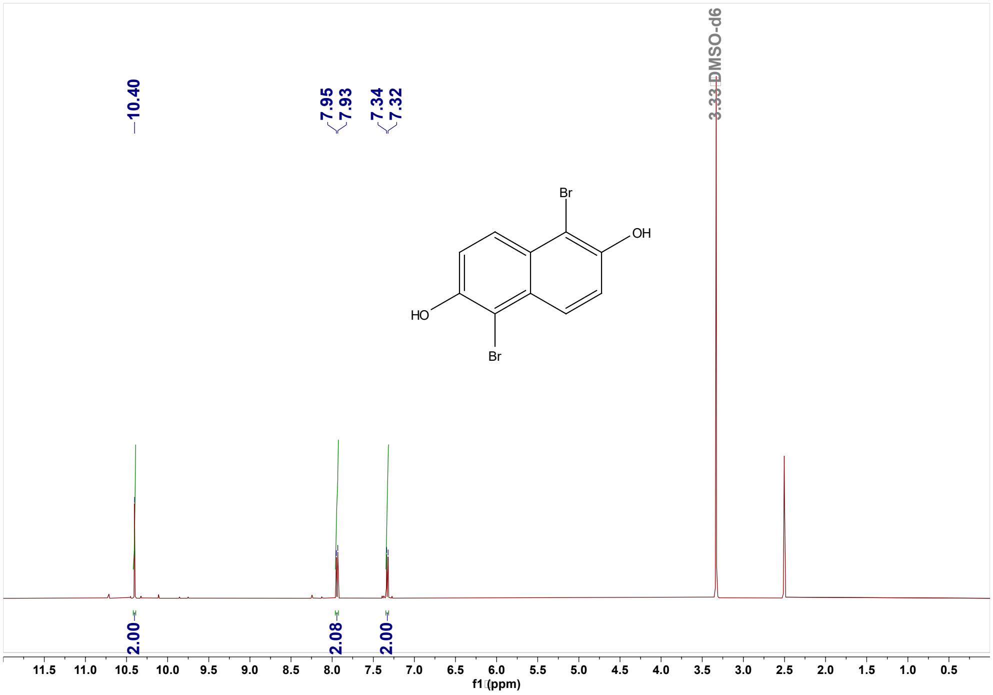

^{1}H NMR spectrum of compound **1a** recorded in DMSO-D$_6$ at room temperature.

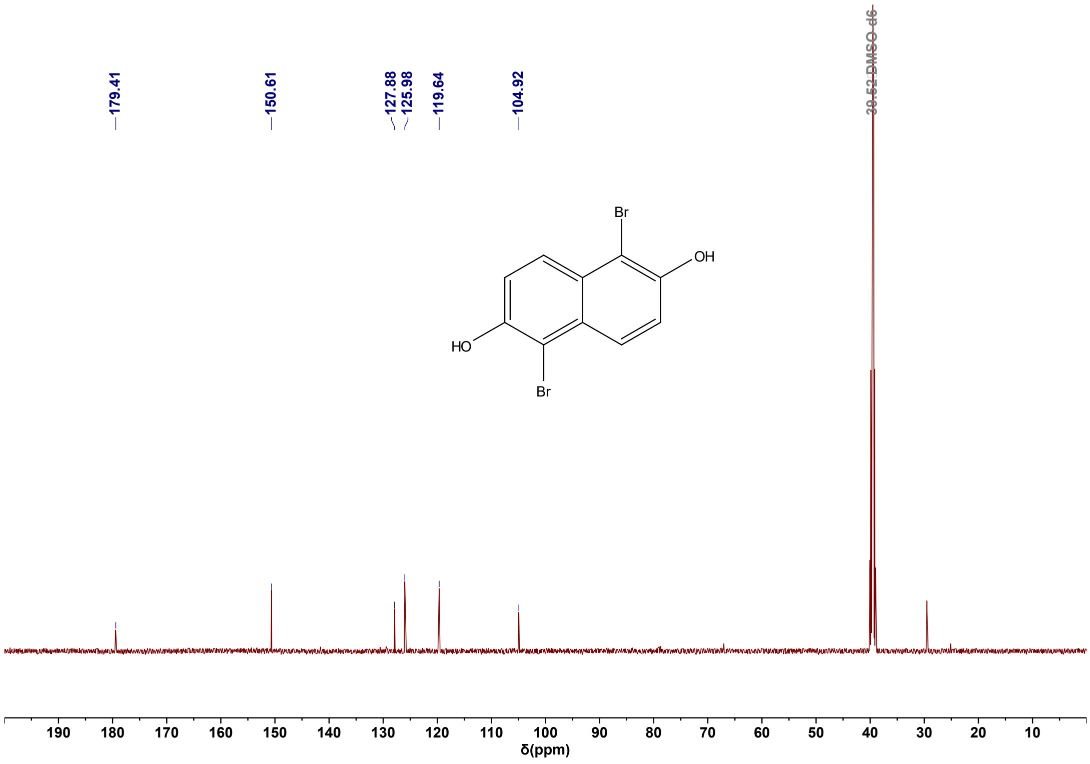

^{13}C NMR spectrum of compound **1a** recorded in DMSO-D$_6$ at room temperature.

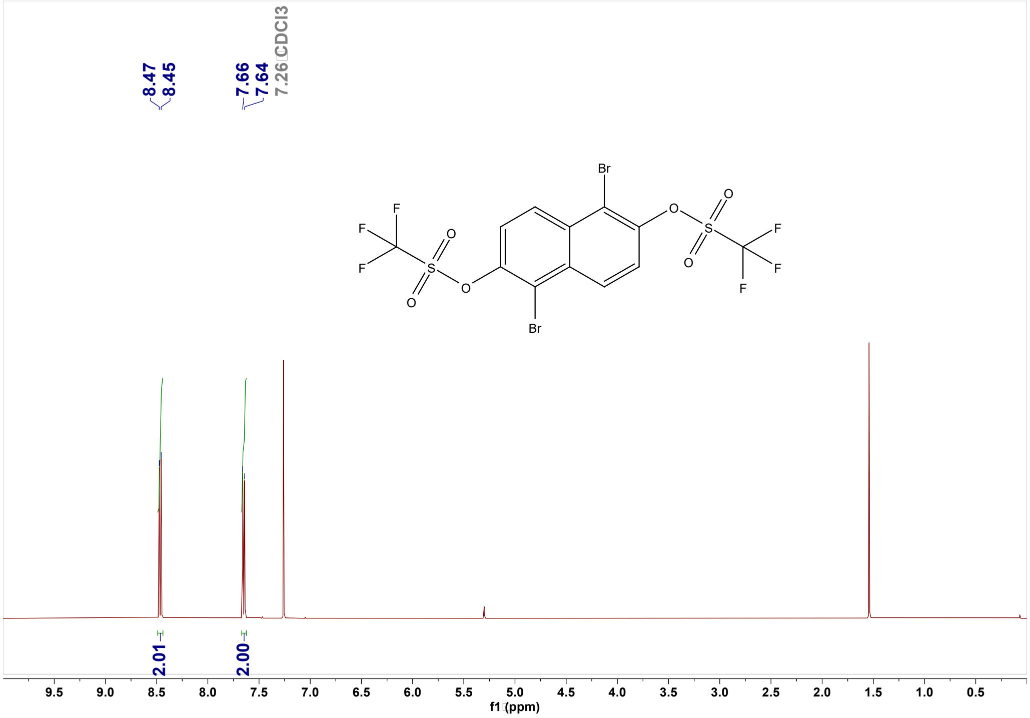

^{1}H NMR spectrum of compound **1b** recorded in $CDCl_3$ at room temperature.

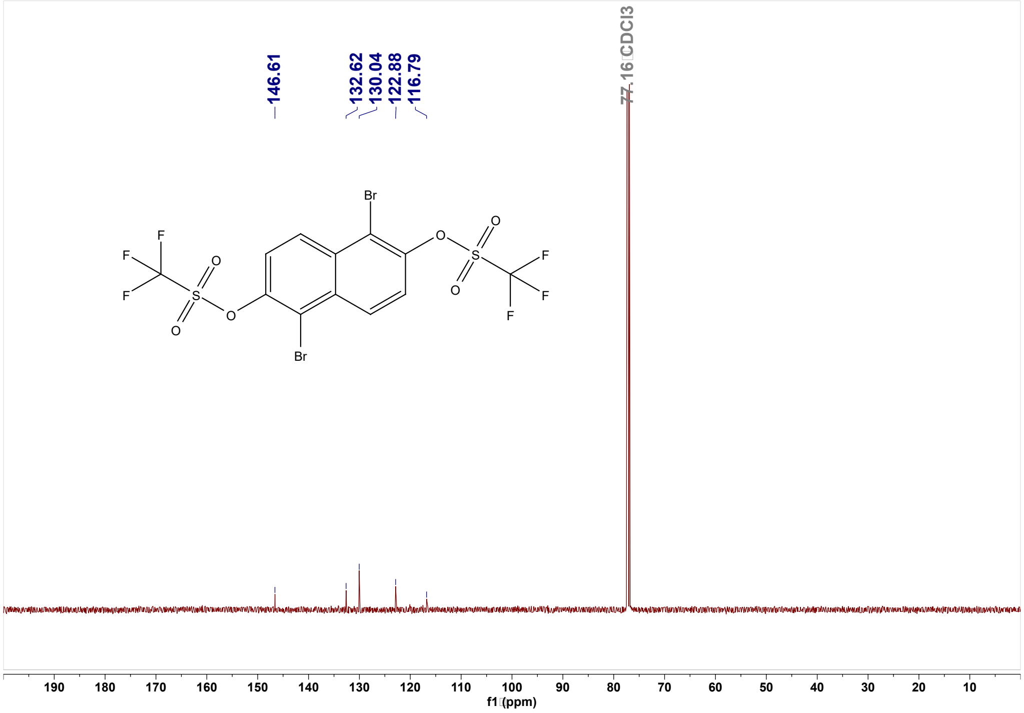

^{13}C NMR spectrum of compound **1b** recorded in $CDCl_3$ at room temperature.

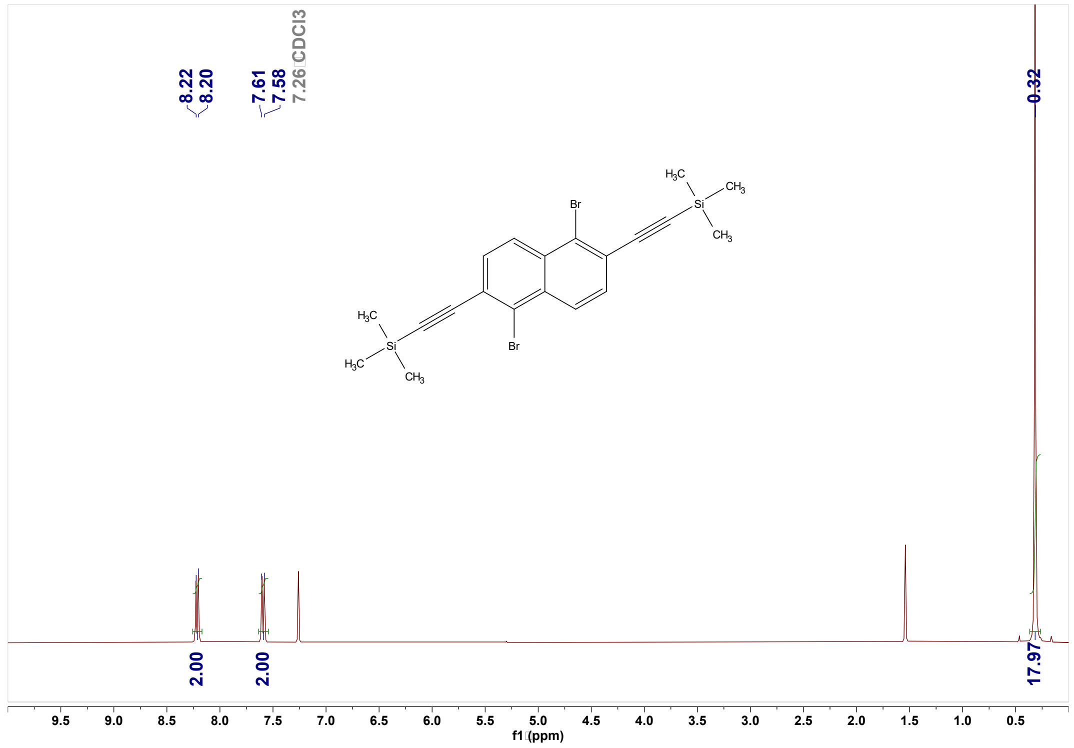

^{1}H NMR spectrum of compound **1c** recorded in $CDCl_3$ at room temperature.

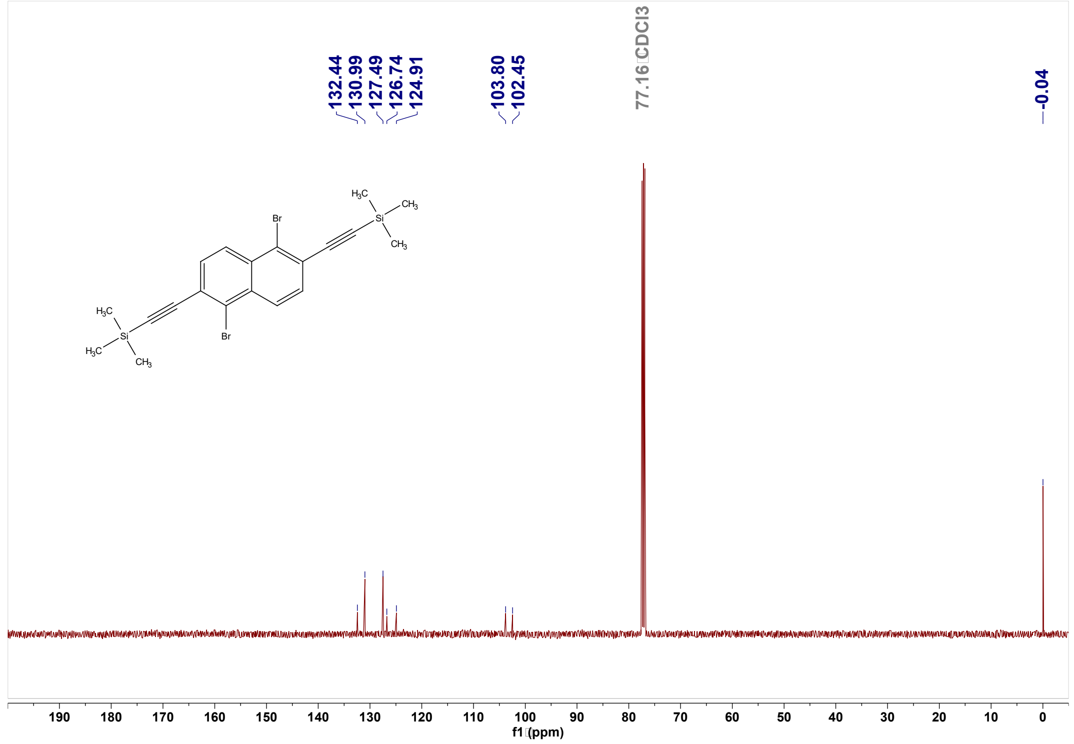

^{13}C NMR spectrum of compound **1c** recorded in $CDCl_3$ at room temperature.

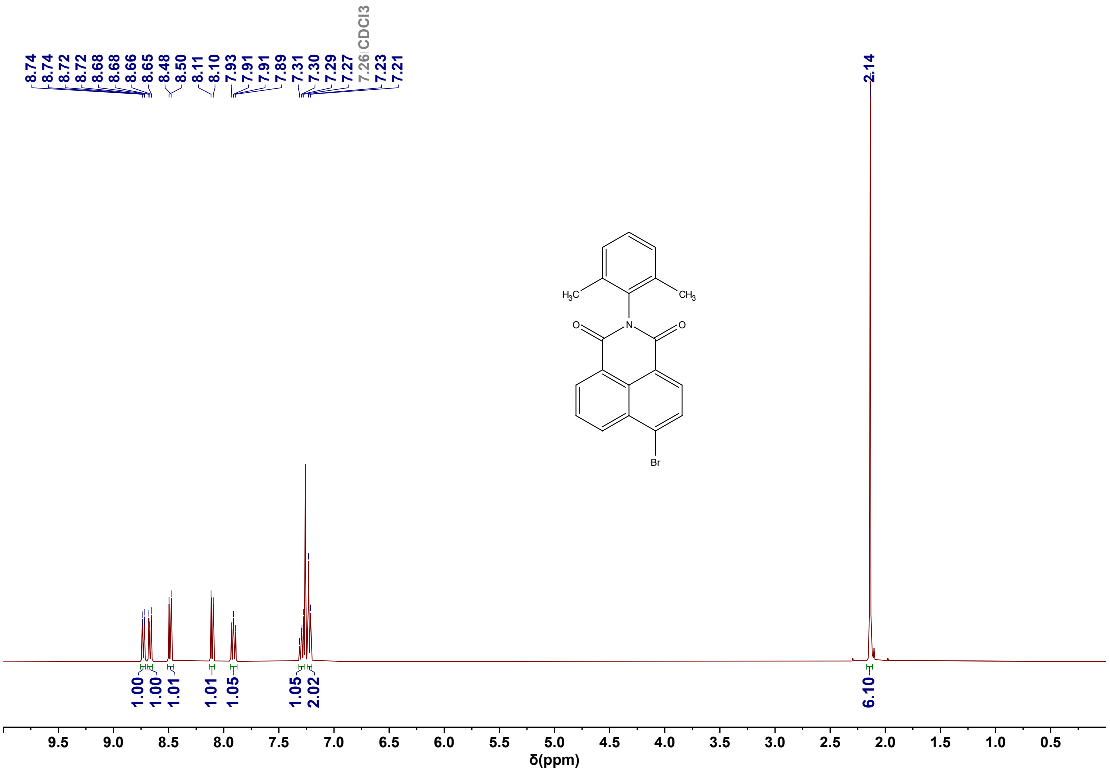

^{1}H NMR spectrum of compound **NMI-Br** recorded in $CDCl_3$ at room temperature.

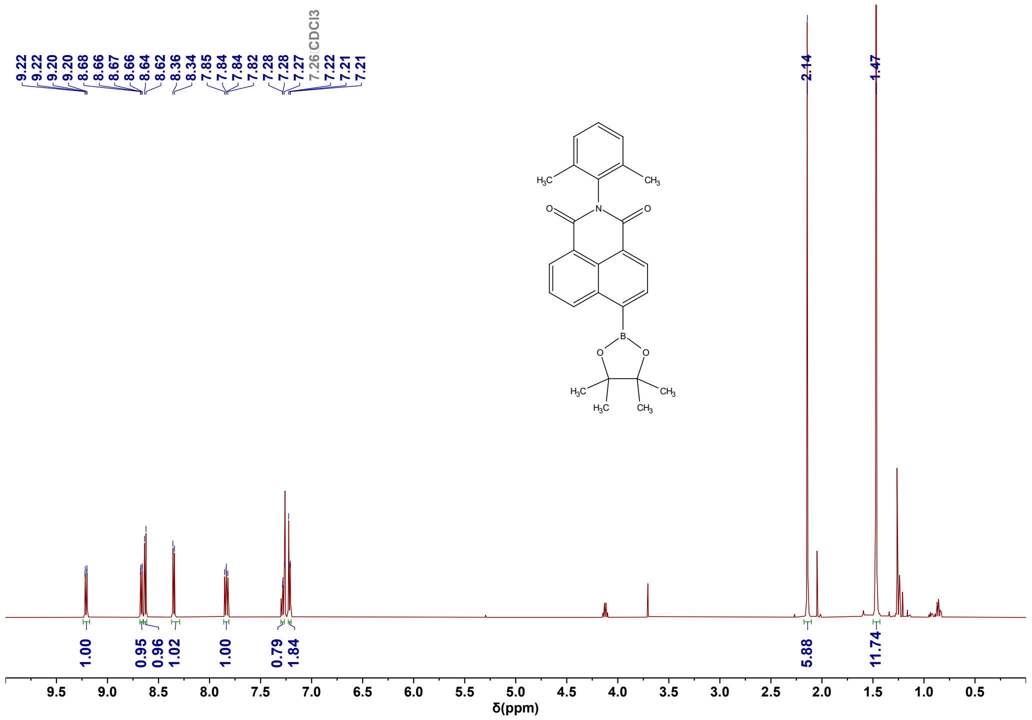

^{1}H NMR spectrum of compound **NMI-Bpin** recorded in $CDCl_3$ at room temperature.

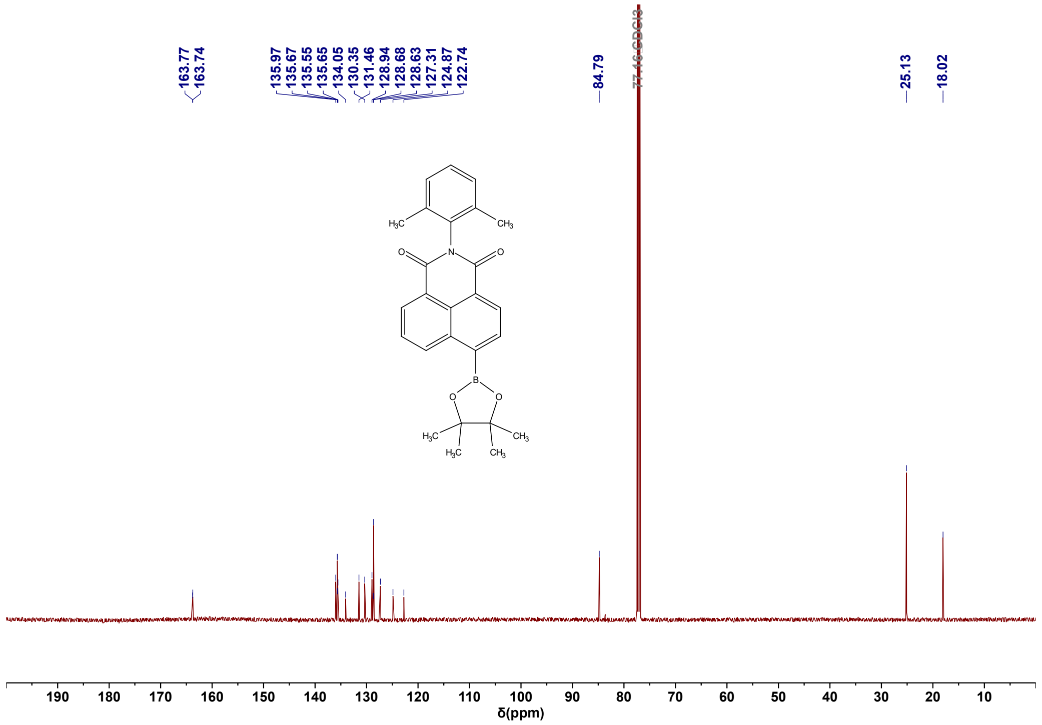

^{13}C NMR spectrum of compound **NMI-Bpin** recorded in $CDCl_3$ at room temperature.

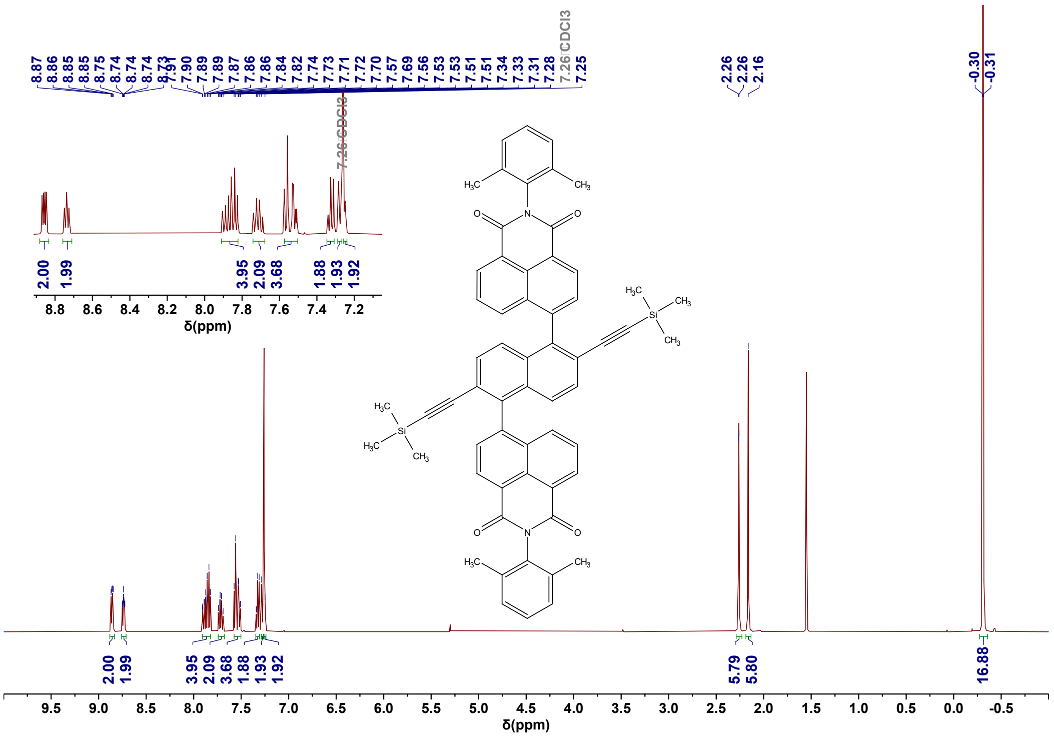

^{1}H NMR spectrum of compound **2** recorded in $CDCl_3$ at room temperature.

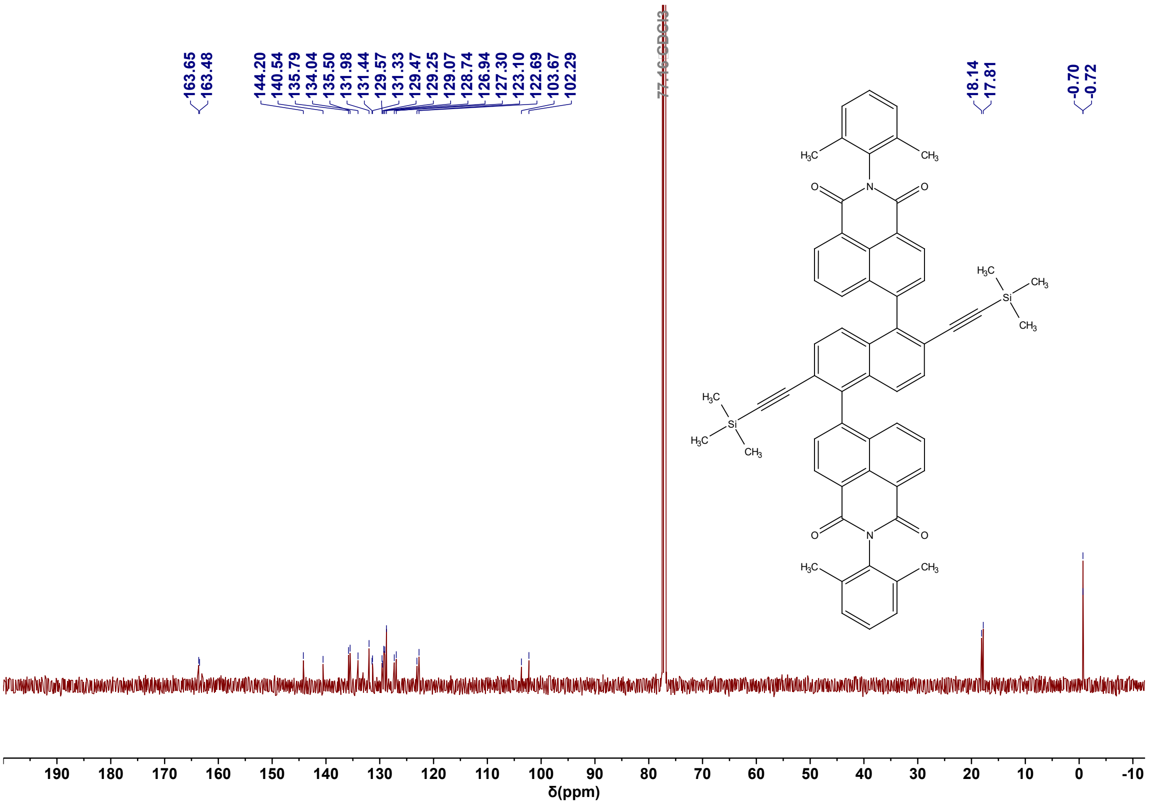

^{13}C NMR spectrum of compound **2** recorded in $CDCl_3$ at room temperature.

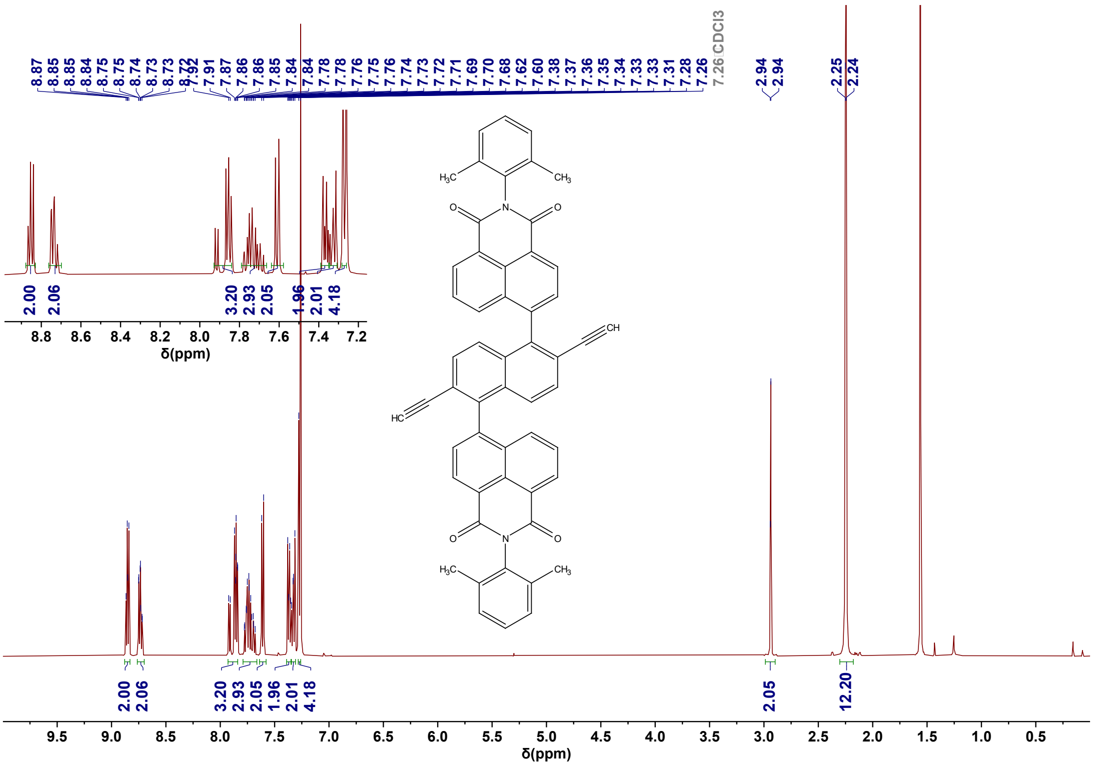

^{1}H NMR spectrum of compound **3** recorded in $CDCl_3$ at room temperature.

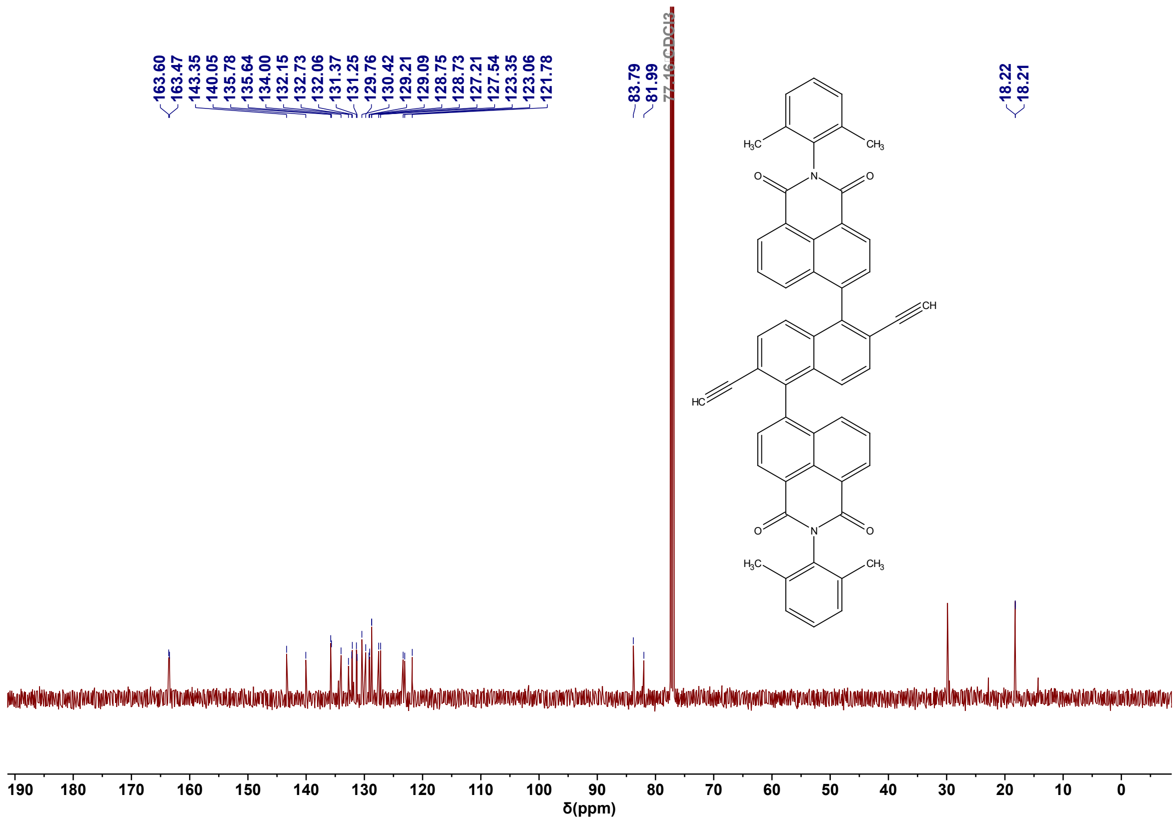

^{13}C NMR spectrum of compound **3** recorded in $CDCl_3$ at room temperature.

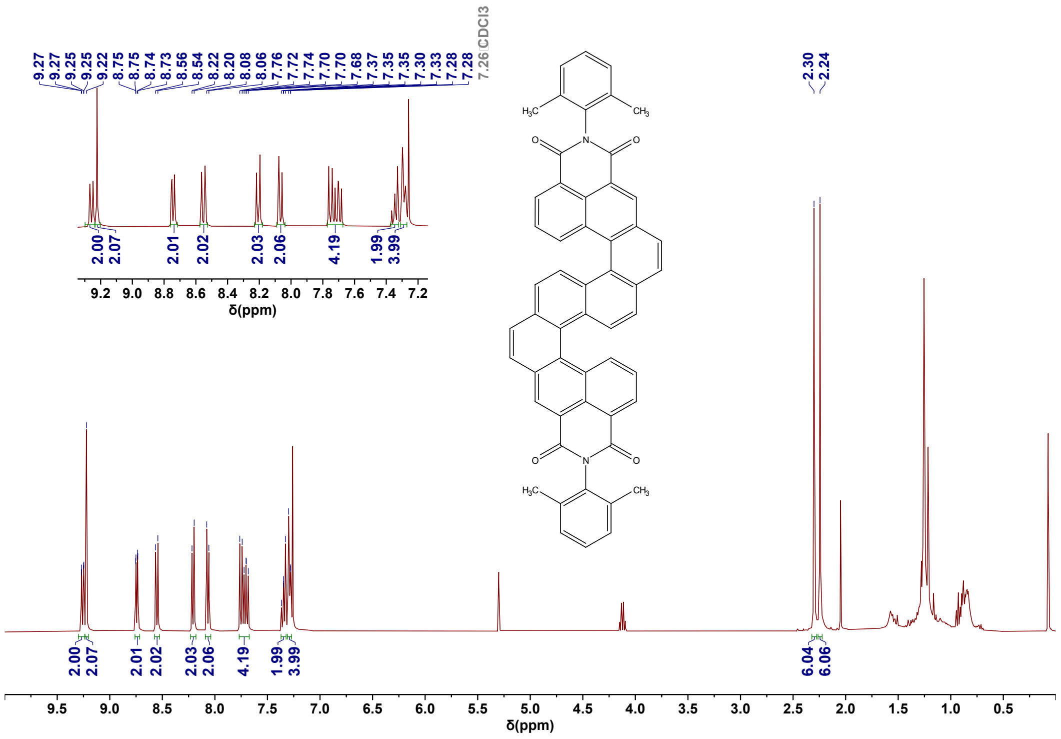

^{1}H NMR spectrum of compound **S-NMI** recorded in $CDCl_3$ at room temperature.

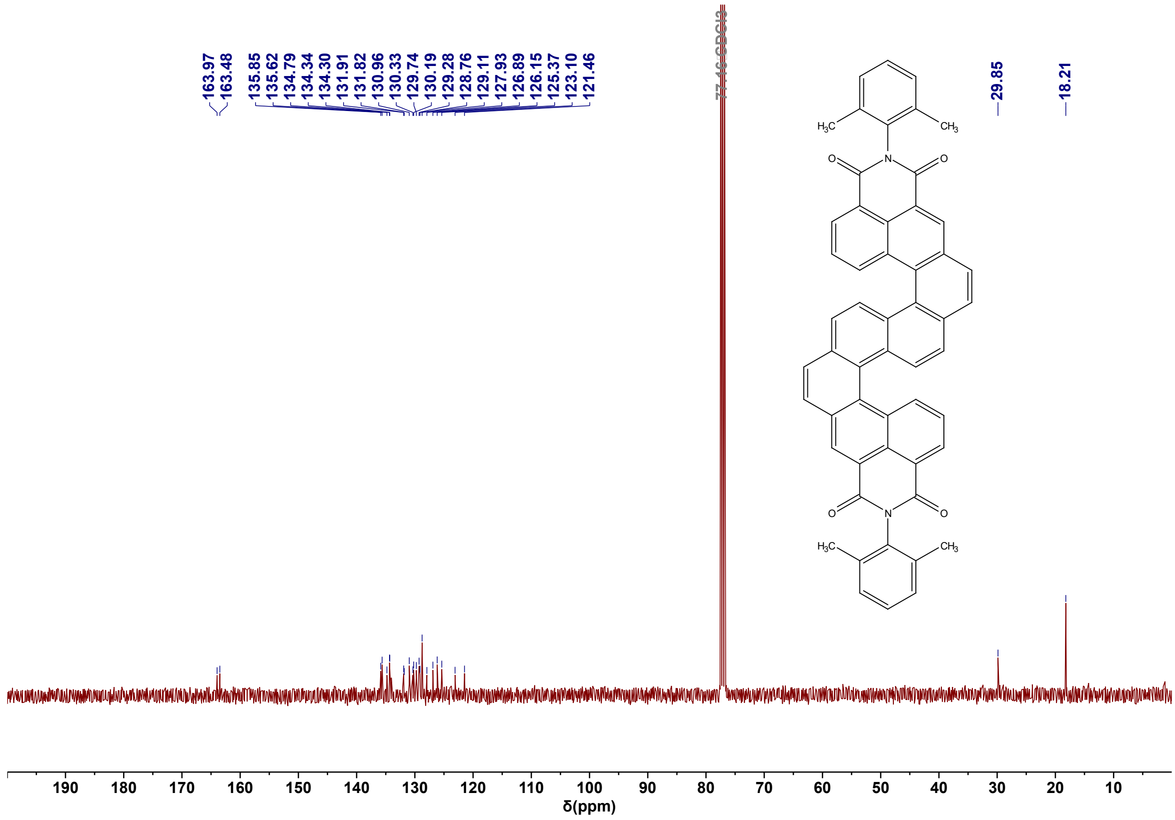

^{13}C NMR spectrum of compound **S-NMI** recorded in $CDCl_3$ at room temperature.

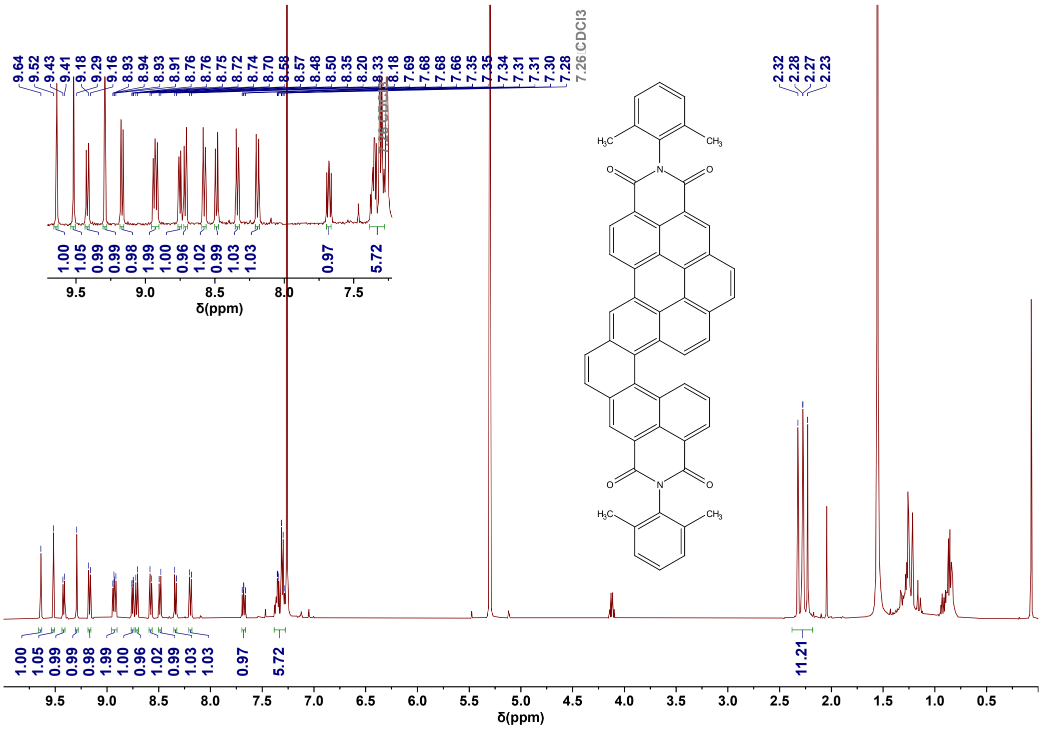

^{1}H NMR spectrum of compound **C-PMI** recorded in $CDCl_3$ at room temperature.

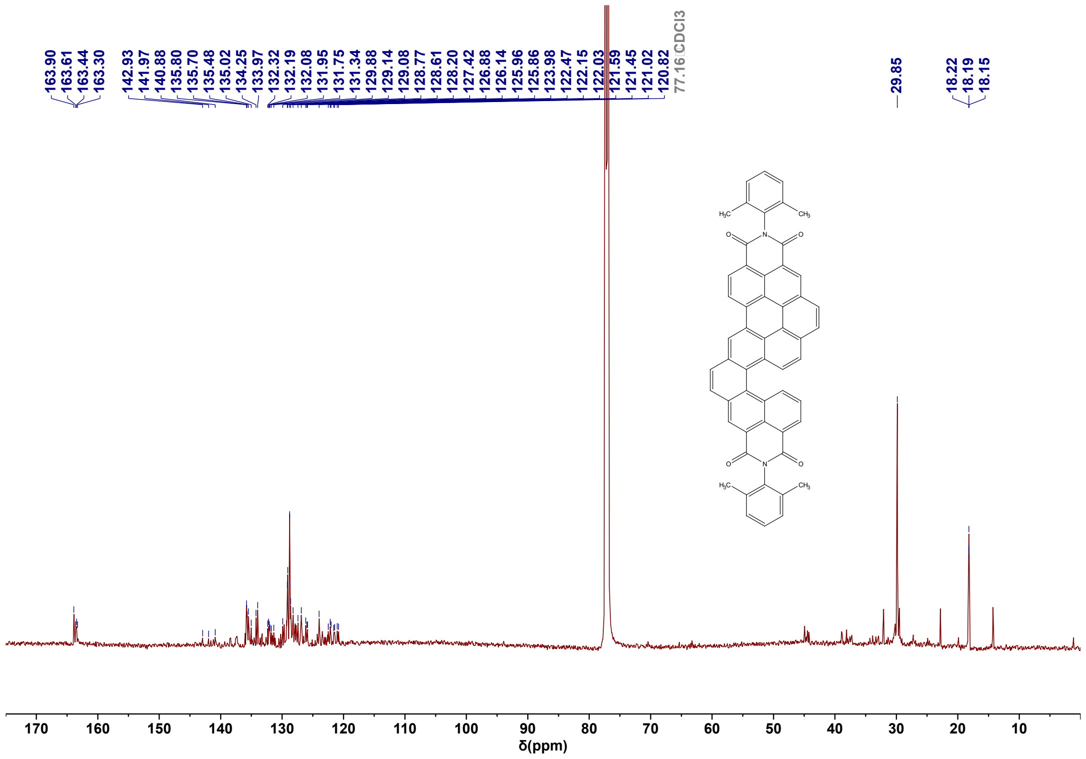

^{13}C NMR spectrum of compound **C-PMI** recorded in $CDCl_3$ at 50 °C.

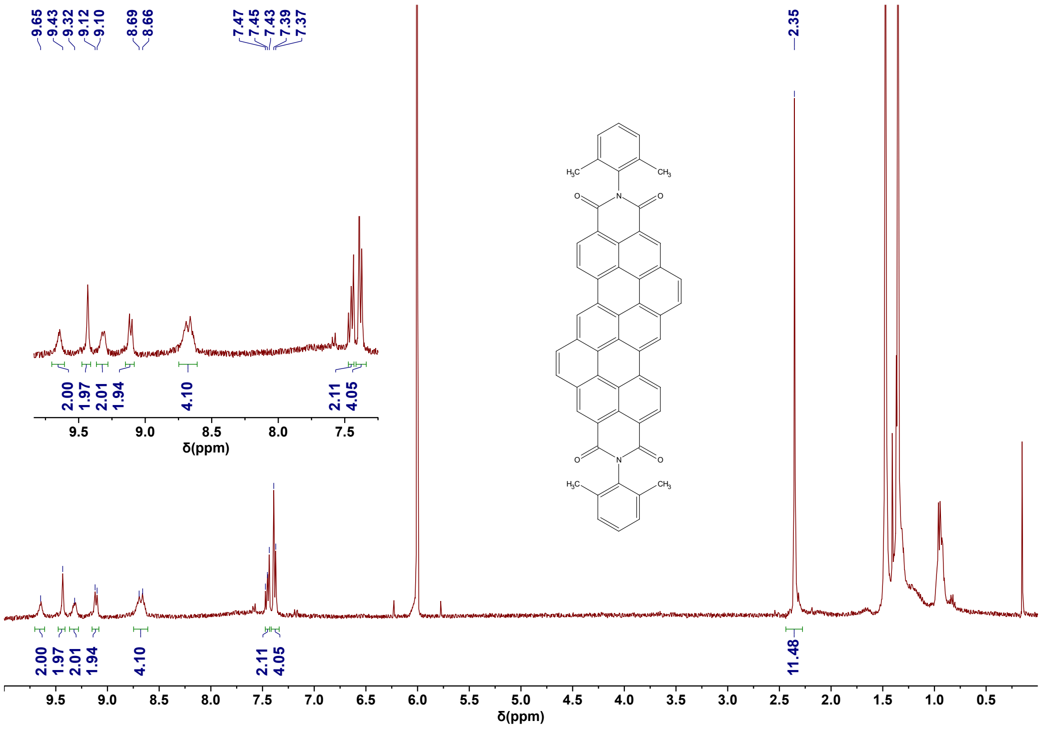

^{1}H NMR spectrum of compound **DB-TDI** recorded in 1,1,2,2-Tetrachloroethane-d_2 at 100 °C.

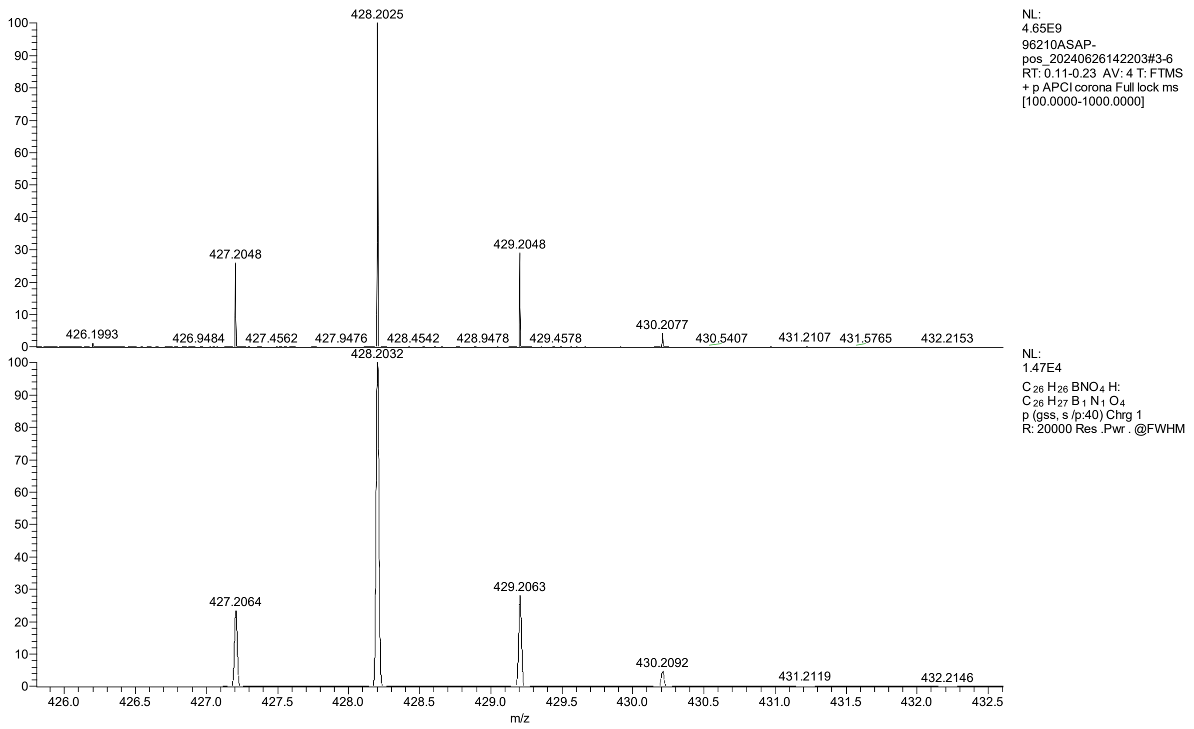

APCI high-resolution mass spectrometry data of **NMI-Bpin.**

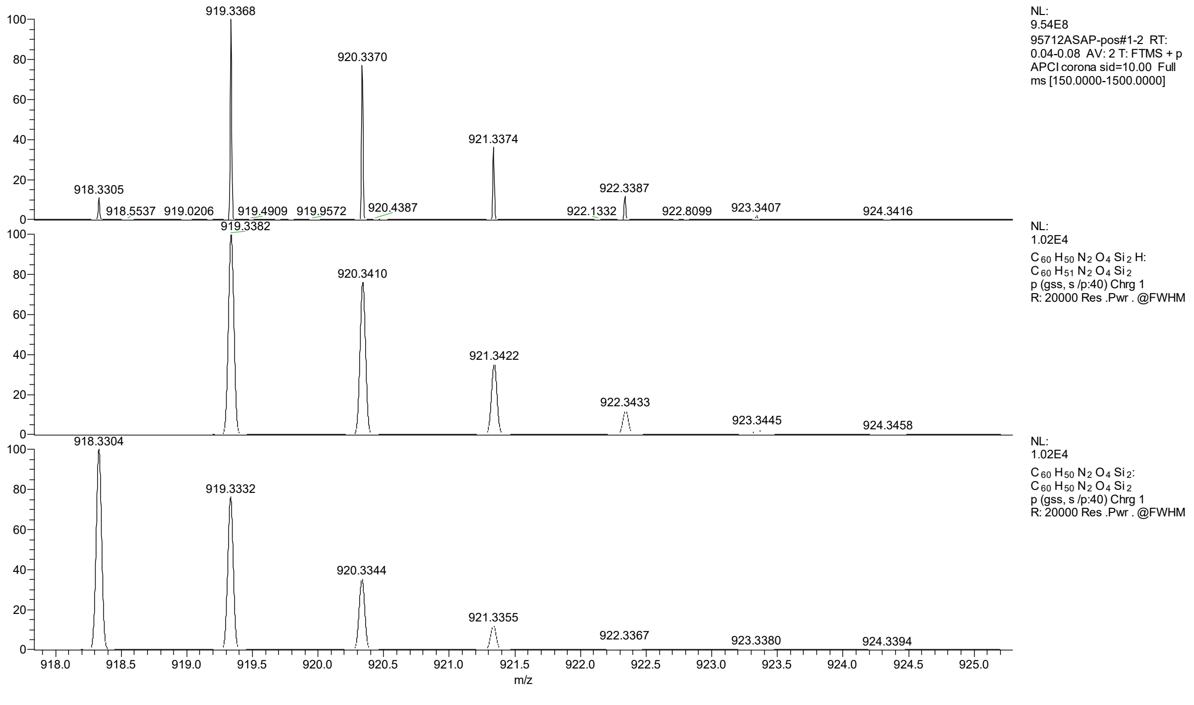

APCI high-resolution mass spectrometry data of compound **2.**

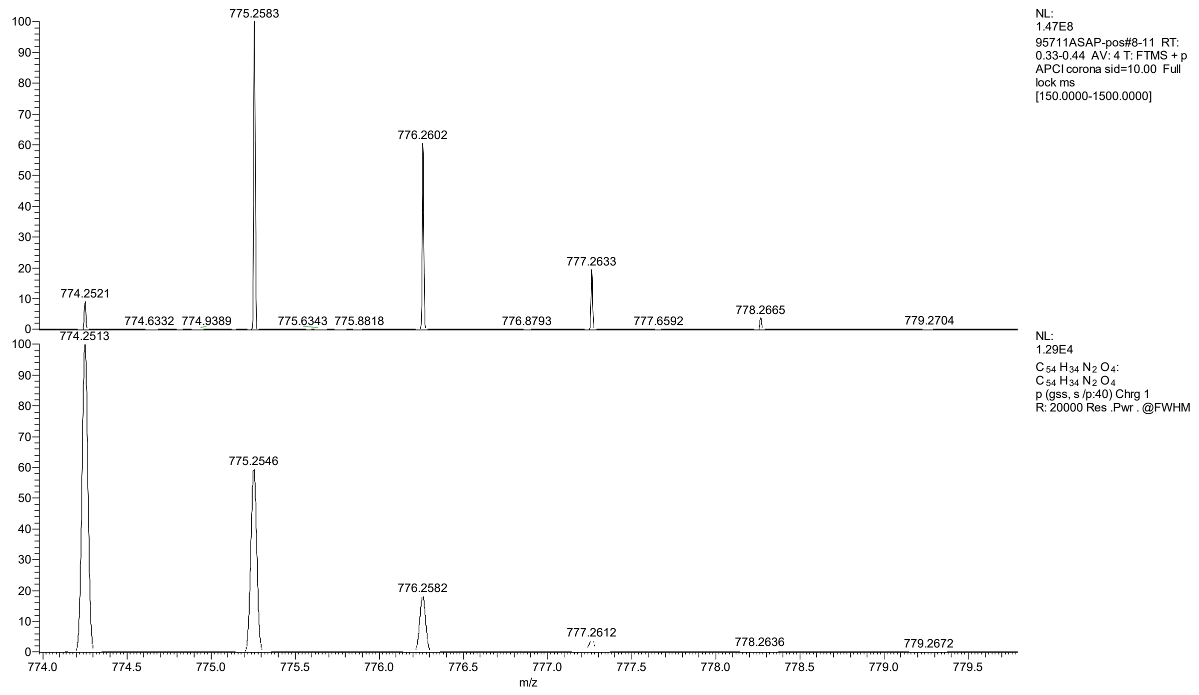

APCI high-resolution mass spectrometry data of compound **3.**

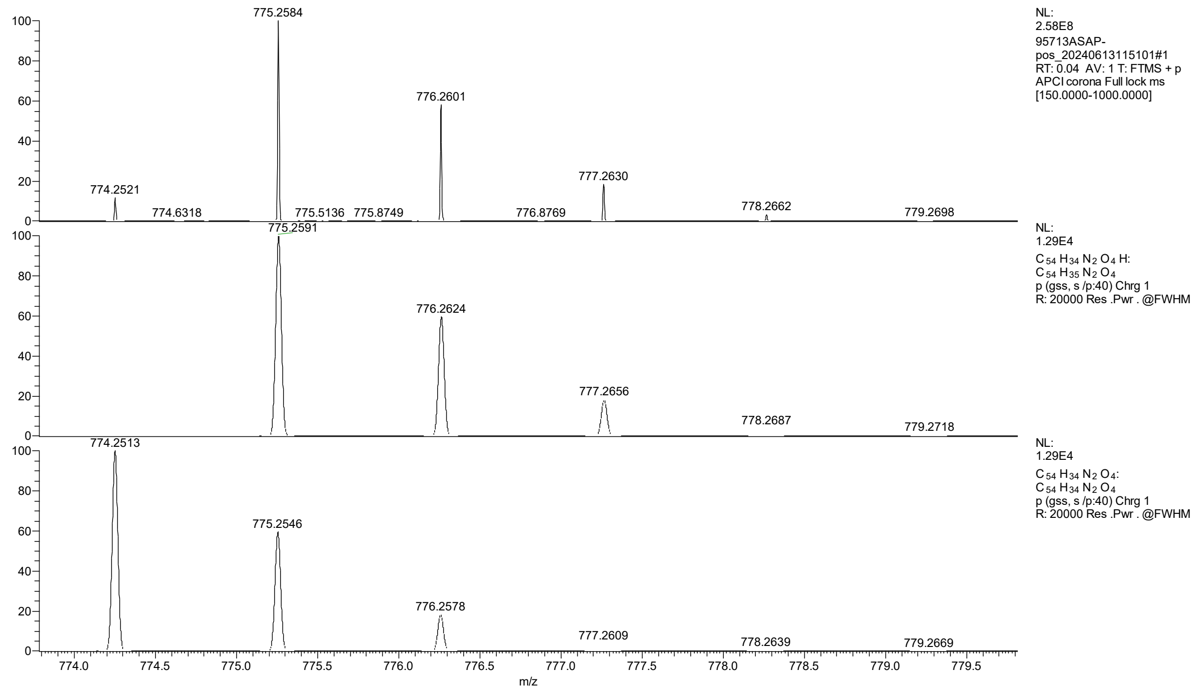

APCI high-resolution mass spectrometry data of **S-NMI.**

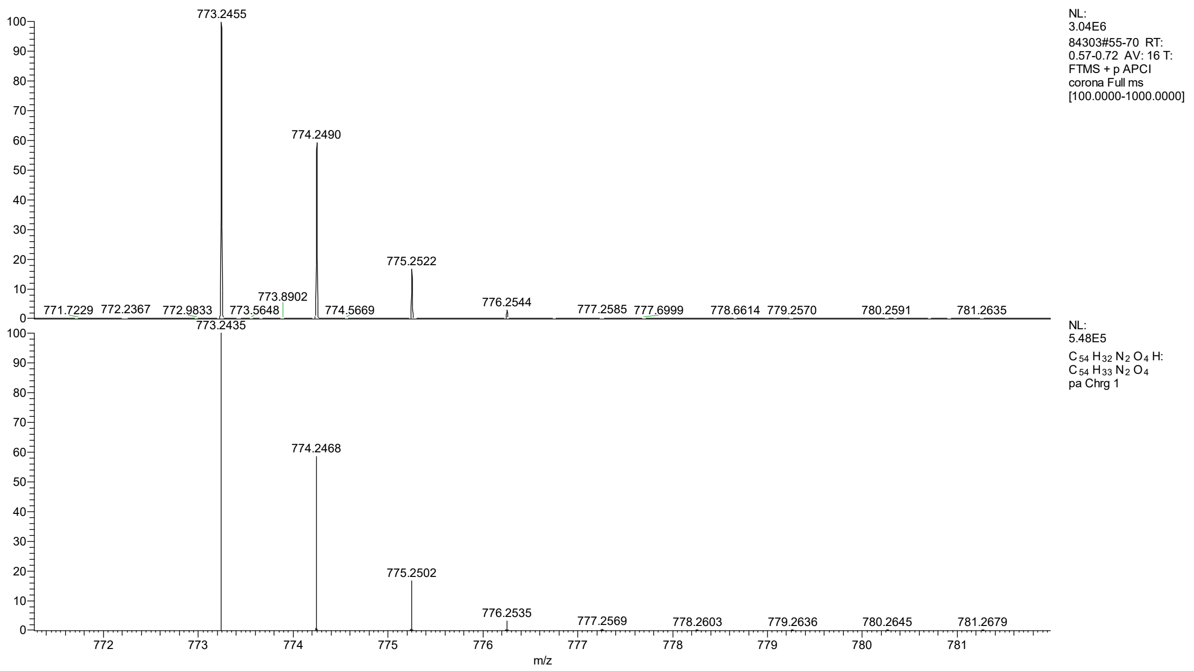

APCI high-resolution mass spectrometry data of **C-PMI.**

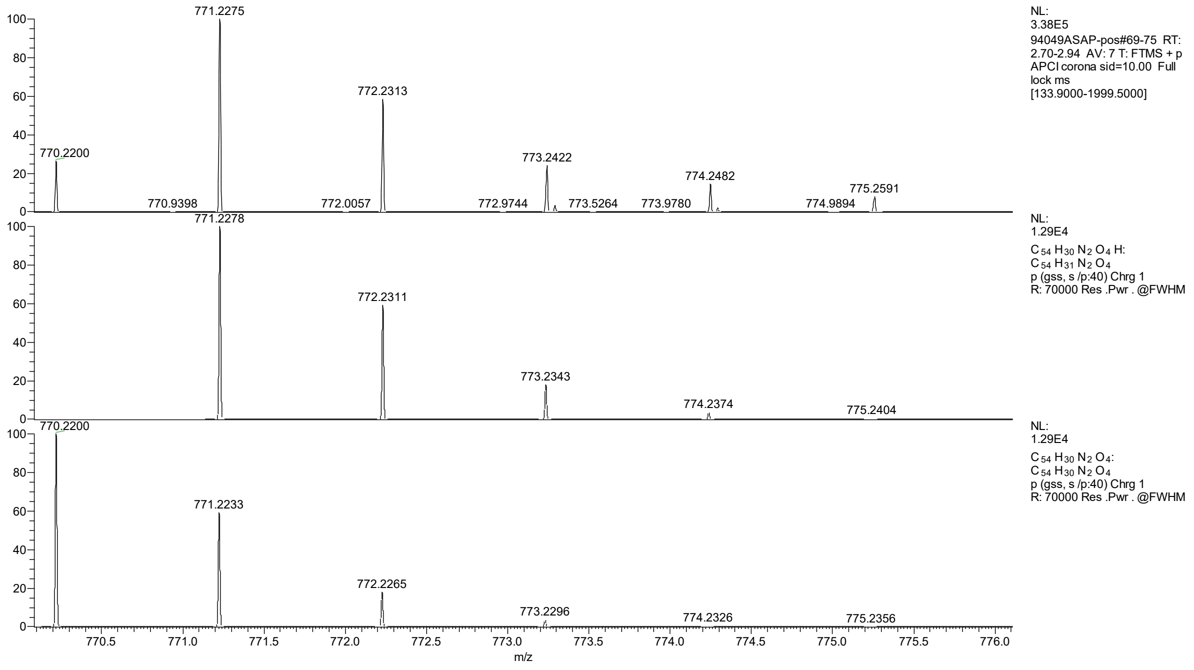

APCI high-resolution mass spectrometry data of **DB-TDI.**

3. Cyclic voltammetry data

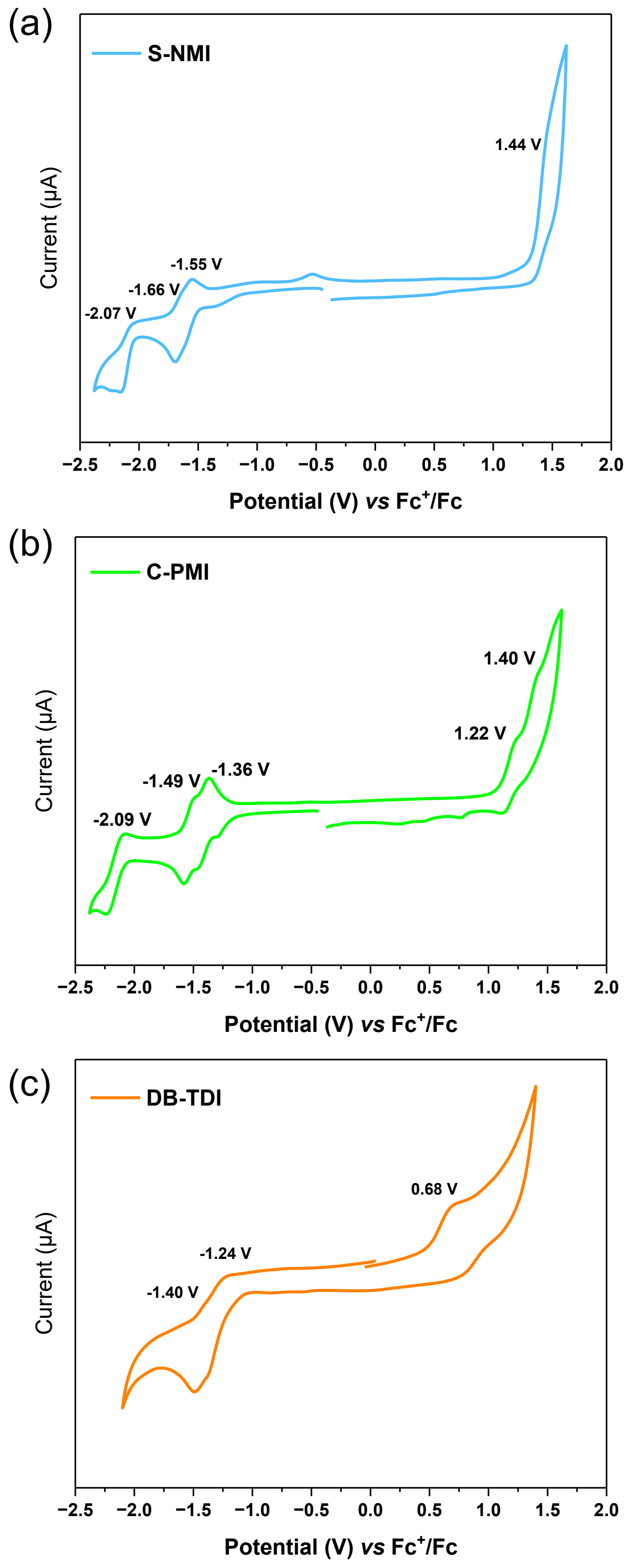

Figure S3. Full CV spectrum for (a) **S-NMI** (b) **C-PMI** (c) **DB-TDI**.

4. Density functional theory (DFT) calculations

DFT were carried out at the B3LYP/6-31G (d) level using Gaussian 09[1] and molecular structures were generated using GaussView 5.0.9[2].

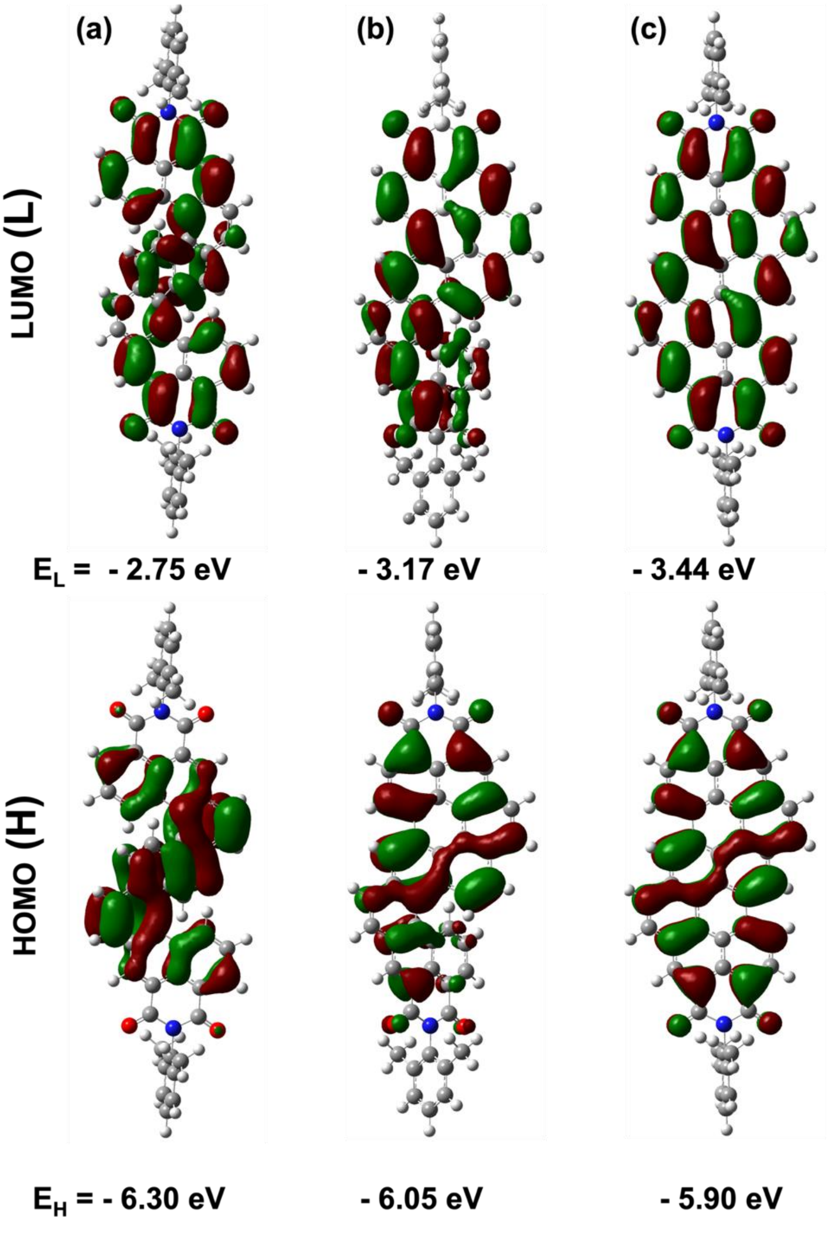

Figure S4. Calculated (B3LYP/6-31G*) HOMO and LUMO profiles of S-NMI (a), C-PMI (b), DB-TDI (c).

5. Crystal data for S-NMI

CCDC number	2451837
Empirical formula	$C_{56}H_{36}Cl_6N_2O_4$
Formula weight	1013.57
Temperature/K	100.01(15)
Crystal system	triclinic
Space group	P-1
a/Å	12.3498(14)
b/Å	13.2971(15)
c/Å	15.7070(18)
α/°	79.729(10)
β/°	67.466(11)
γ/°	86.799(9)
Volume/Å^3	2344.0(5)
Z	2
ρ_{calc}g/cm^3	1.436
μ/mm^{-1}	3.759
F(000)	1040.0
Crystal size/mm^3	0.532 × 0.299 × 0.076
Radiation	Cu Kα (λ = 1.54184)
2Θ range for data collection/°	6.182 to 151.632
Index ranges	$-15 \leq h \leq 15$, $-16 \leq k \leq 16$, $-19 \leq l \leq 18$
Reflections collected	36264
Independent reflections	9425 [R_{int} = 0.0355, R_{sigma} = 0.0226]
Data/restraints/parameters	9425/0/617
Goodness-of-fit on F^2	1.056
Final R indexes [I>=2σ (I)]	R_1 = 0.0469, wR_2 = 0.1233
Final R indexes [all data]	R_1 = 0.0482, wR_2 = 0.1244
Largest diff. peak/hole / e Å^{-3}	1.01/-0.93

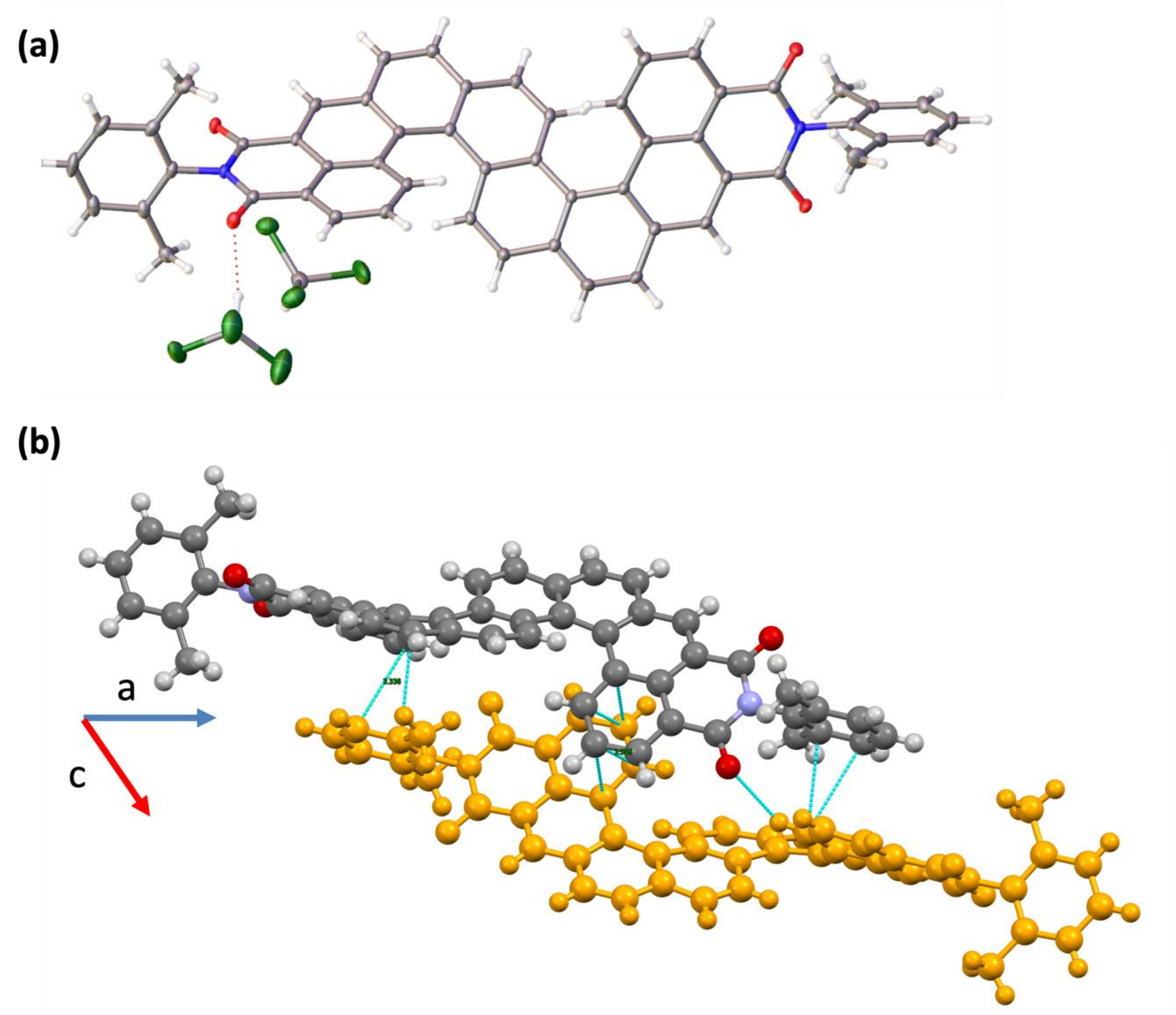

Figure S5. (a) Structure of **S-NMI** in the crystal. Dark green = chlorine, red = oxygen, blue = nitrogen, grey = carbon, white = hydrogen. Atomic displacement parameters displayed with 50% probability. (b) Crystal structure showing the dimer of **S-NMI** enantiomers in the *ac* plane, with each enantiomer molecule highlighted in a different color for clarity.

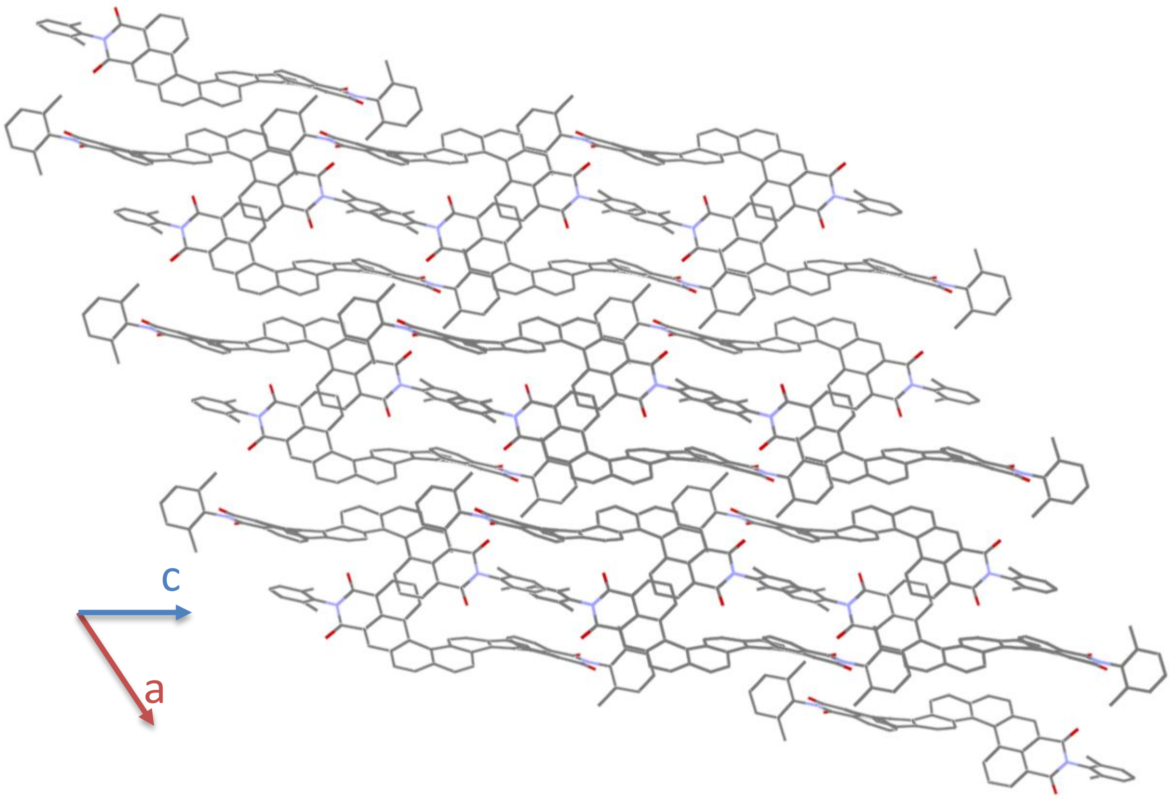

Figure S6. (a) Crystal packing pattern of **S-NMI** enantiomers in the *ac* plane.

6. Time-resolved photoluminescence data

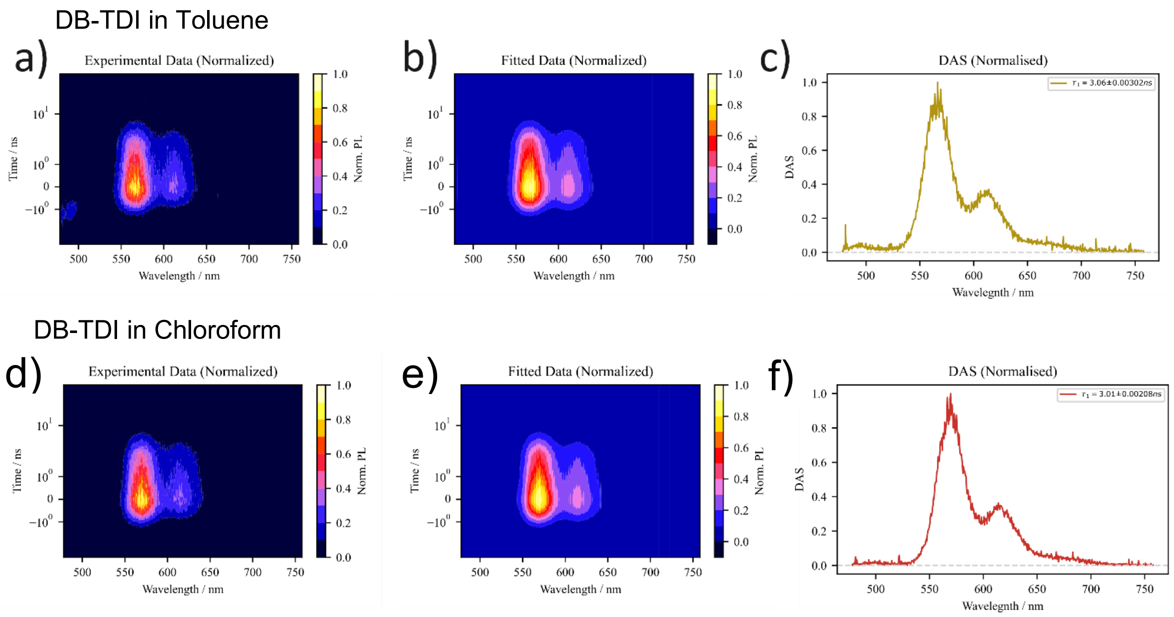

Figure S7. Global analysis of time-resolved photoluminescence data in both toluene and chloroform for **DB-TDI**. **a, d)** Experimental 2D time-resolved emission plots. **b, e)** Corresponding fitted data obtained from global analysis. **c, f)** Decay-associated spectra (DAS) extracted from the fits, showing distinct spectral components and their associated lifetimes (τ).

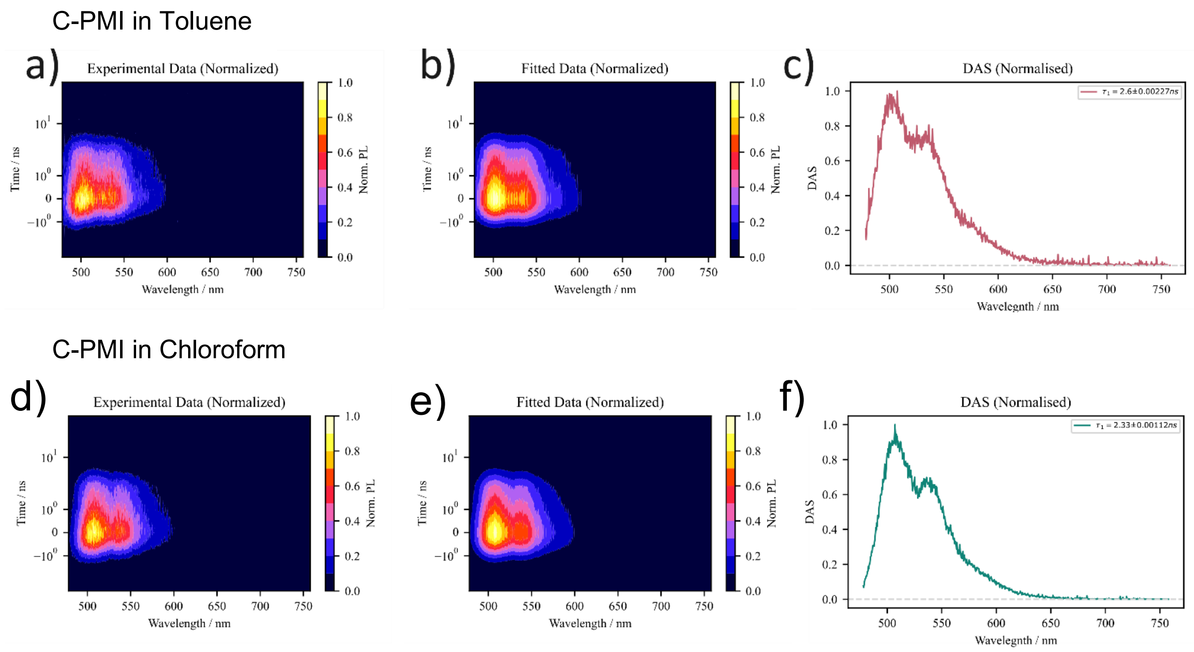

Figure S8. Global analysis of time-resolved photoluminescence data in both toluene and chloroform for **C-PMI**. **a, d)** Experimental 2D time-resolved emission plots. **b, e)** Corresponding fitted data obtained from global analysis. **c, f)** Decay-associated spectra (DAS) extracted from the fits, showing distinct spectral components and their associated lifetimes (τ).

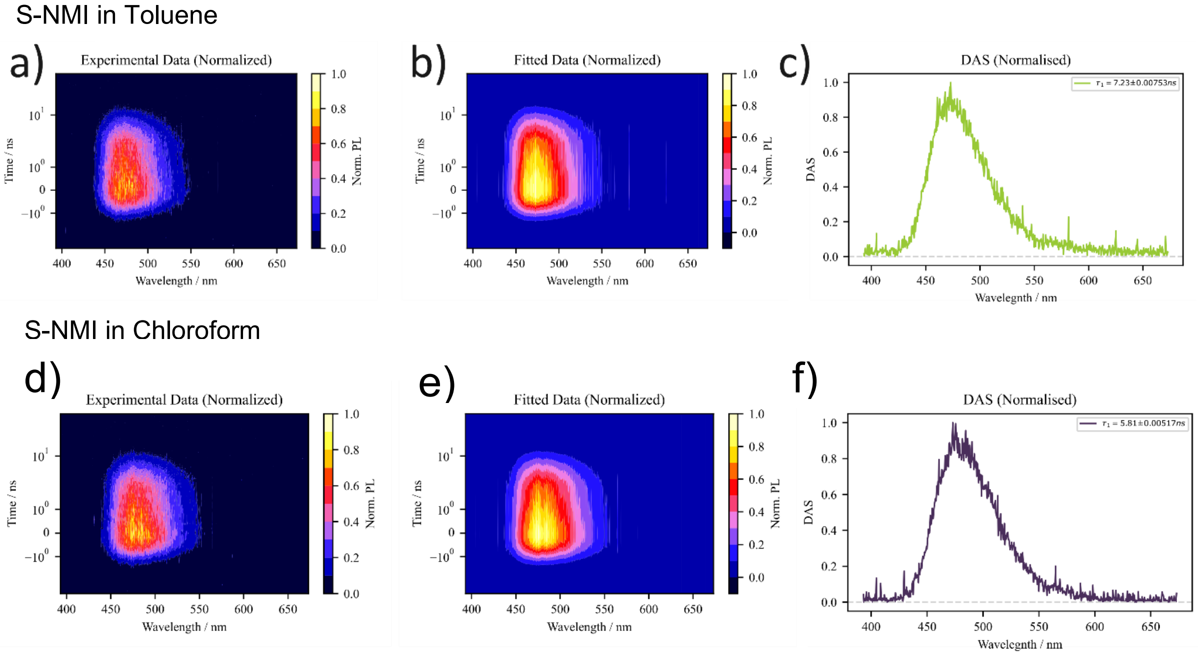

Figure S9. Global analysis of time-resolved photoluminescence data in both toluene and chloroform for **S-NMI**. **a, d)** Experimental 2D time-resolved emission plots. **b, e)** Corresponding fitted data obtained from global analysis. **c, f)** Decay-associated spectra (DAS) extracted from the fits, showing distinct spectral components and their associated lifetimes (τ).

7. Grazing Incidence Wide Angle Scattering Data

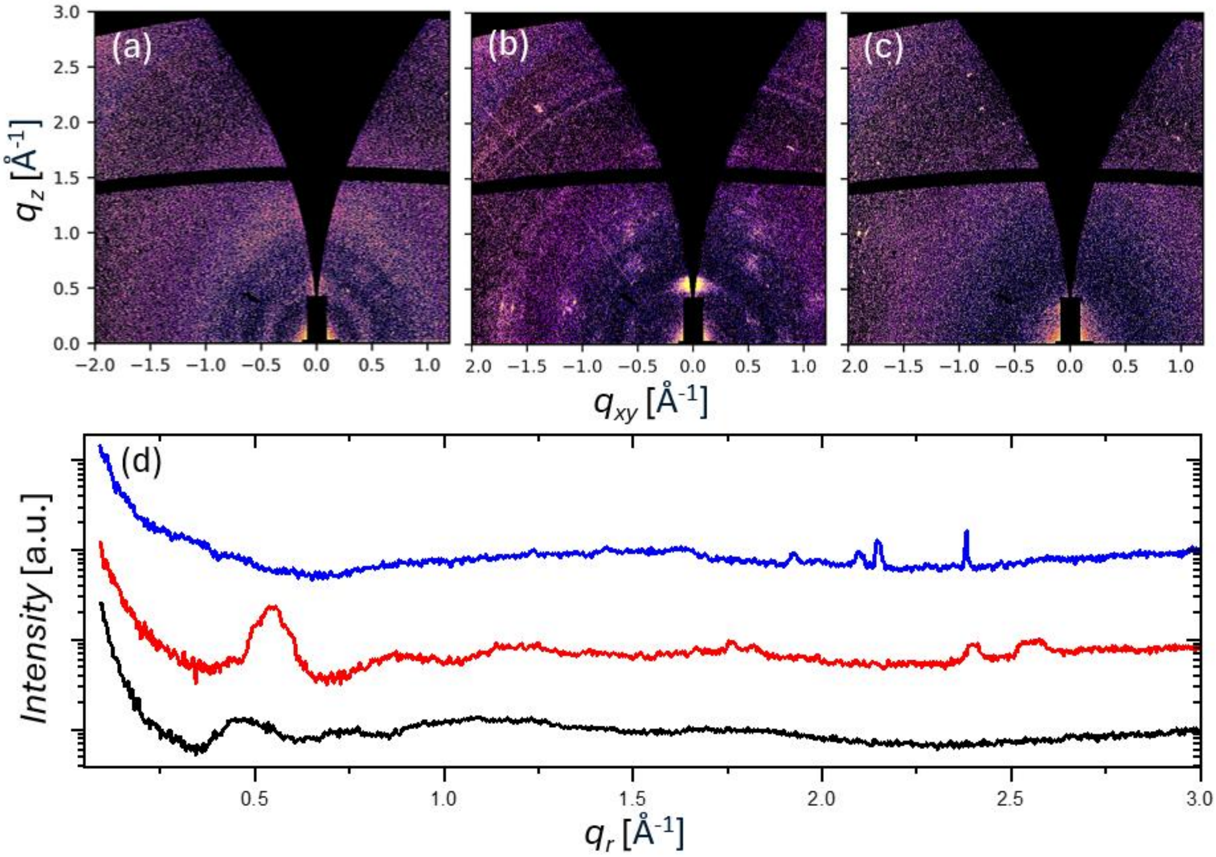

Figure S10. Two Grazing Incidence Wide Angle X-ray Scattering data (a) **S-NMI** (b) **C-PMI** (c) **DB-TDI** and radially integrated scattering data (d), where **S-NMI** (black) **C-PMI** (red) **DB-TDI** (blue).

8. Chiral HPLC Chromatogram

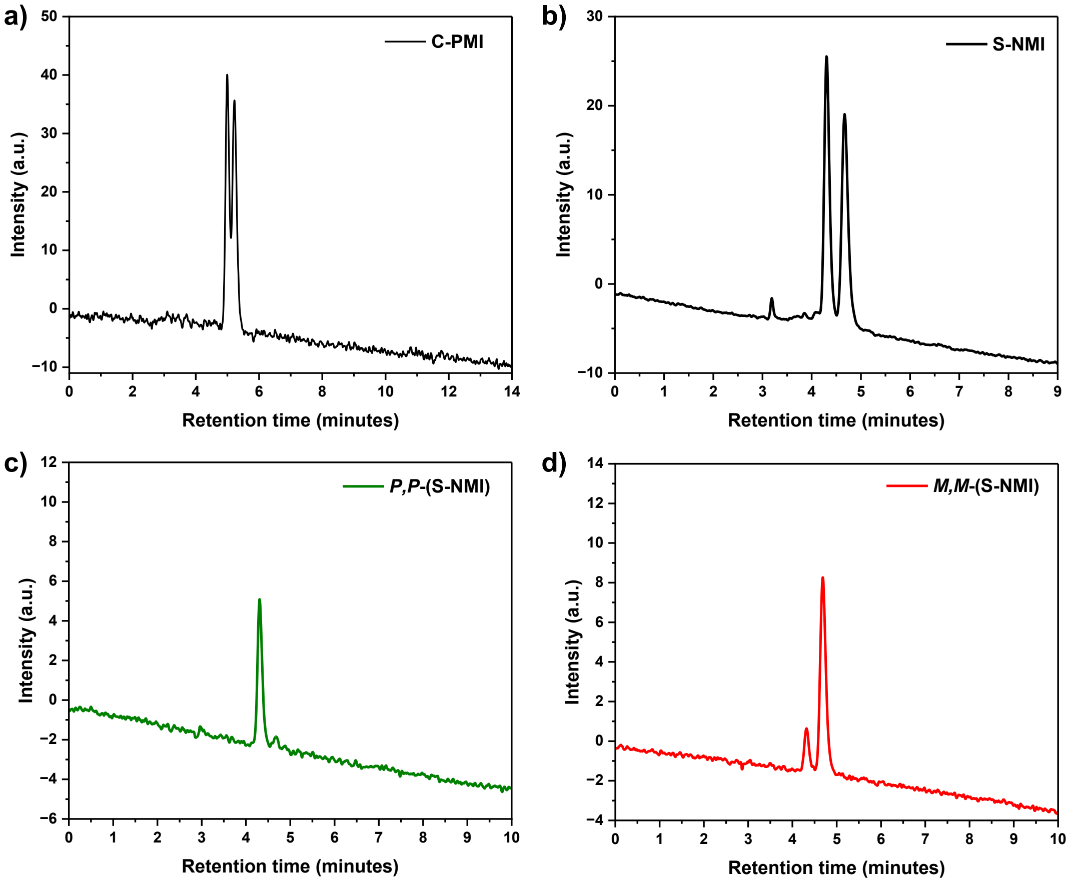

Figure S11. Chromatograms of a) **C-PMI** (racemates); b) **S-NMI** (racemates); c) ***P,P*-(S-NMI)** and **d) *M,M*-(S-NMI).** Chiral separations were conducted using Lux 5 um i-Amylose-3 LC column (250 mm × 21.1 mm). Eluent for **S-NMI**: CH_2Cl_2/n-hexane = 90/10 (*v/v*), **C-PMI**: CH2Cl2/n-hexane = 90/20 (*v/v*), with 15 mL/min flowrate.

9. References

[1] G. W. T. M. J. Frisch, H. B. Schlegel, G. E. Scuseria, M. A. Robb, J. R. Cheeseman, G. Scalmani, V. Barone, B. Mennucci, G. A. Petersson, H. Nakatsuji, M. Caricato, X. Li, H. P. Hratchian, A. F. Izmaylov, J. Bloino, G. Zheng, J. L. Sonnenberg, M. Hada, M. Ehara, K. Toyota, R. Fukuda, J. Hasegawa, M. Ishida, T. Nakajima, Y. Honda, O. Kitao, H. Nakai, T. Vreven, J. A. Montgomery, Jr., J. E. Peralta, F. Ogliaro, M. Bearpark, J. J. Heyd, E. Brothers, K. N. Kudin, V. N. Staroverov, R. Kobayashi, J. Normand, K. Raghavachari, A. Rendell, J. C. Burant, S. S. Iyengar, J. Tomasi, M. Cossi, N. Rega, J. M. Millam, M. Klene, J. E. Knox, J. B. Cross, V. Bakken, C. Adamo, J. Jaramillo, R. Gomperts, R. E. Stratmann, O. Yazyev, A. J. Austin, R. Cammi, C. Pomelli, J. W. Ochterski, R. L. Martin, K. Morokuma, V. G. Zakrzewski, G. A. Voth, P. Salvador, J. J. Dannenberg, S. Dapprich, A. D. Daniels, O. Farkas, J. B. Foresman, J. V. Ortiz, J. Cioslowski, and D. J. Fox, Vol. Revision D.01 (Ed.: W. CT), Gaussian, Inc., 2013.

[2] R. Dennington, T. Keith, J. Millam, Semichem Inc., Shawnee Mission KS, GaussView, Version 5, 2009.
